# Cytoskeleton-dependent clustering of membrane-bound prion protein on the cell surface

**DOI:** 10.1016/j.jbc.2021.100359

**Published:** 2021-02-02

**Authors:** Stefanie Hackl, Xue Wen Ng, Danqin Lu, Thorsten Wohland, Christian F.W. Becker

**Affiliations:** 1 Institute of Biological Chemistry, Faculty of Chemistry, University of Vienna, Vienna, Austria; 2 Departments of Biological Sciences and Chemistry and Centre for Bioimaging Sciences (CBIS), National University of Singapore (NUS), Singapore; 3 School of Chemistry and Molecular Engineering, East China Normal University, Shanghai, China

**Keywords:** prion protein, protein aggregation, membrane-associated proteins, membrane biophysics, cytoskeleton, fluorescence correlation spectroscopy, protein semisynthesis, ACF, autocorrelation function, CBD, chitin-binding domain, CCF, cross-correlation function, CD, circular dichroism, CDI, carbonyldiimidazole, DCM, dichloromethane, EGFR, epidermal growth factor receptor, EPL, expressed protein ligation, EMCCD, electron multiplying charge-coupled device, ESI-MS, electrospray ionization–mass spectrometry, FCS, fluorescence correlation spectroscopy, GPI, glycosylphosphatidylinositol, HBSS, Hanks' Balanced Salt Solution, ICA, intensity correlation analysis, ICQ, intensity correlation quotient, ITIR-FCS, imaging total internal reflection–FCS, mβCD, methyl-β-cyclodextrin, PCC, Pearson's correlation coefficient, PrP^Sc^, scrapie prion protein, PrP^C^, cellular prion protein, PSF, point spread function, ROI, region of interest, RP-HPLC, reversed-phase high-performance liquid chromatography, SDS-PAGE, sodium dodecyl sulfate–polyacrylamide gel electrophoresis, SPPS, solid-phase peptide synthesis, SR-SIM, superresolution structured illumination microscopy, TCA, trichloroacetic acid, TIS, triisopropylsilane, TSE, transmissible spongiform encephalopathy

## Abstract

Prion diseases are a group of neurodegenerative disorders that infect animals and humans with proteinaceous particles called prions. Prions consist of scrapie prion protein (PrP^Sc^), a misfolded version of the cellular prion protein (PrP^C^). During disease progression, PrP^Sc^ replicates by interacting with PrP^C^ and inducing its conversion to PrP^Sc^. Attachment of PrP^C^ to cellular membranes *via* a glycosylphosphatidylinositol (GPI) anchor is critical for the conversion of PrP^C^ into PrP^Sc^. However, the mechanisms governing PrP^C^ conversion and replication on the membrane remain largely unclear. Here, a site-selectively modified PrP variant equipped with a fluorescent GPI anchor mimic (PrP-GPI) was employed to directly observe PrP at the cellular membrane in neuronal SH-SY5Y cells. PrP-GPI exhibits a cholesterol-dependent membrane accumulation and a cytoskeleton-dependent mobility. More specifically, inhibition of actin polymerization reduced the diffusion of PrP-GPI indicating protein clustering, which resembles the initial step of PrP aggregation and conversion into its pathogenic isoform. An intact actin cytoskeleton might therefore prevent conversion of PrP^C^ into PrP^Sc^ and offer new therapeutic angles.

Prion diseases or transmissible spongiform encephalopathies (TSEs) are incurable, neurodegenerative disorders (1). The central pathophysiologic event is ascribed to the conformational change of the α-helical, cellular (PrP^C^) into the toxic, β-sheet-enriched scrapie prion protein (PrP^Sc^) (2, 3). PrP^Sc^ can then not only propagate further PrP^C^ misfolding but is also capable of infecting other organisms (4). To date, the mechanisms of PrP^C^-PrP^Sc^ conversion, PrP^Sc^ replication, and the molecular pathways leading to neurodegeneration are largely unknown. Native PrP^C^ is tethered *via* its glycosylphosphatidylinositol (GPI) anchor to the outer leaflet of neuronal plasma membranes (5). Several studies ascribe a crucial role in the pathogenesis of prion diseases and PrP^C^-PrP^Sc^ conversion to membrane attachment of PrP (6, 7, 8, 9, 10, 11, 12, 13), where contact between endogenous PrP^C^ and exogenous PrP^Sc^ can easily occur and membrane properties can impact protein structure and function.

In this context, deciphering the earliest pathophysiologic events in prion diseases by direct observation of posttranslationally modified PrP at the cellular membrane, the initial site of prion infection and PrP misfolding is of utmost importance. So far, most studies used recombinant PrP lacking posttranslational modifications as a surrogate (14, 15, 16, 17, 18) or heterogeneous protein preparations isolated from mammalian cell lines (14, 15, 16). To this end, semisynthesis offers a unique possibility to access homogeneous, membrane-anchored, labeled PrP-GPI (Fig. 1). The semisynthetic approach relies on linking a synthetic membrane anchoring peptide to recombinantly produced PrP-α-thioester by expressed protein ligation (EPL) (17, 18, 19). We have previously applied similar strategies to generate a variety of modified PrP variants (20, 21, 22, 23). These experiments revealed specific binding of PrP-GPI to membranes, distinct from unmodified PrP, with impact on biochemical and conformational properties of the protein. Critical roles for both the *C*-terminal and the *N*-terminal domain of PrP-GPI were found (22). Noteworthy, PrP containing a GPI anchor mimic performed similar as PrP equipped with a native GPI anchor (20, 21). Here, we aim at directly studying the movement of semisynthetic membrane-anchored PrP in its native environment by fluorescence microscopy. This setup allows discerning the effect of membrane properties on PrP mobility and their role in triggering protein misfolding.

**Figure 1:**
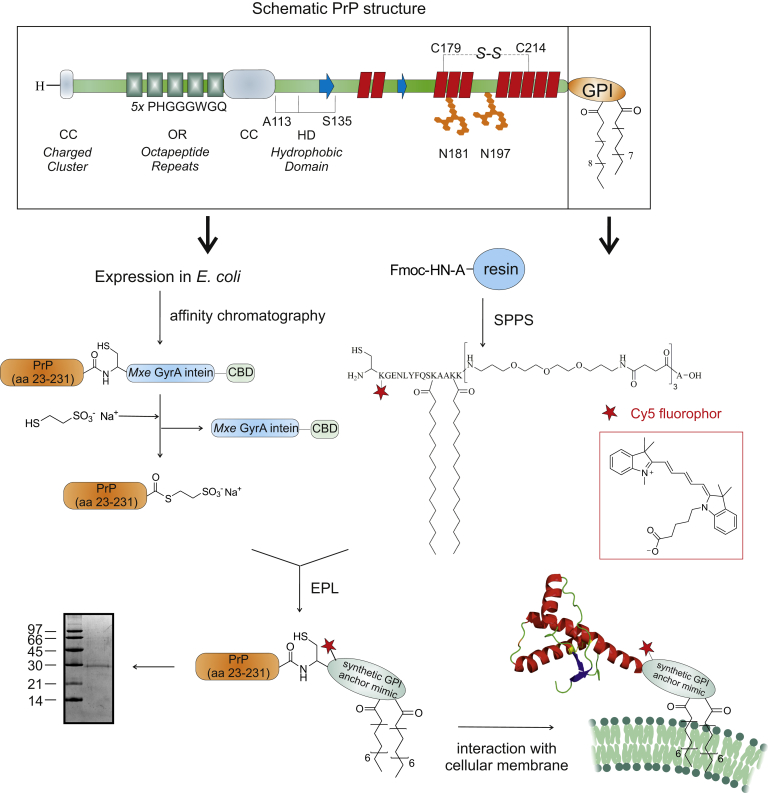
**Semisynthesis strategy for directly studying the interaction between PrP and the cellular membrane by using Cy5-labeled PrP (aa 23–231) equipped with a GPI anchor mimic (PrP-GPI).** Schematic outline of the primary (top) and the tertiary structure of PrP^C^ is based on NMR measurements and residue numbers of human PrP^C^ (aa 23–230). The tertiary structure of PrP^C^ was taken from the protein data bank (PDB) entry 1QLZ (77).

## Results

### Semisynthesis of homogeneously Cy5-labeled PrP equipped with a GPI anchor mimic (PrP-GPI)

Extension of our semisynthetic approach toward additional fluorescence labeling with Cy5 produced homogeneously labeled PrP-GPI to directly study the PrP–cellular membrane interaction within living neuronal SH-SY5Y cells (Fig. 1). Expressed protein ligation (EPL) under optimized conditions afforded pure PrP-GPI with a yield of 14% over all steps (ESI), which was folded with 69% yield. CD spectroscopy revealed a predominantly α-helical fold, in very good agreement with previously reported results (Fig. S1) (22, 24, 25).

### PrP-membrane localization studied by structured illumination microscopy (SIM) imaging

**Figure 2:**
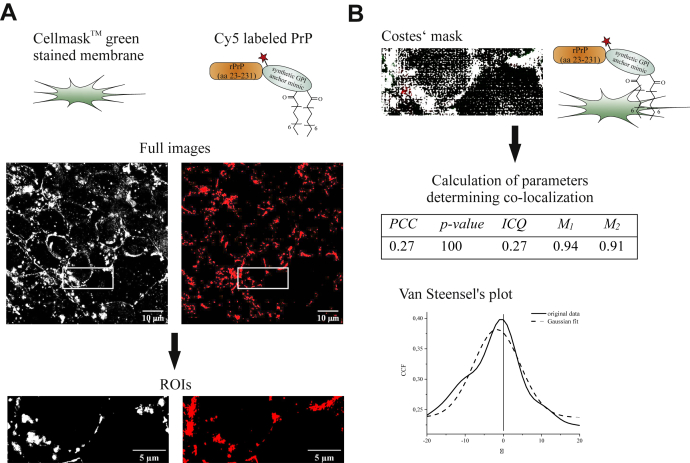
**SR images and colocalization analysis of SH-SY5Y cells incubated with the Cy5-labeled PrP-GPI.***A*, SR images and the corresponding ROIs (regions of interest) used in the colocalization analysis show the CellMask green (*left*) and Cy5 fluorescence (*right*) of the membrane and PrP-GPI. *B*, a maximum intensity Z-projection of the Costes' mask (*white*: colocalization, *black*: background, *red*: Cy5 fluorescence, *green*: CellMask green fluorescence) illustrates the colocalization based on calculated thresholds according to Costes' statistical significance algorithm (29). Based on that, the colocalization was quantified with calculated intensity correlation quotients (ICQ) (64), Pearson's (PCC) and Manders' coefficients (M_1_, M_2_) (27, 28), and statistically evaluated with *p*-values by Costes' (29). Additional evidence for colocalization was provided by the plot of the Van Steensel's cross-correlation functions (CCFs) (31).

In turn, cell imaging experiments based on superresolution structured illumination microscopy (SR-SIM) combined with colocalization analysis were then applied to study the cellular localization of folded Cy5-labeled PrP-GPI. In our experimental setup, superresolution (SR) images of SH-SY5Y cells were acquired with SIM (Fig. 2*A*).

Prior to fixation, these cells were incubated for 1 h with Cy5-labeled PrP-GPI in the presence of Cellmask (to stain membranes). In order to accelerate the process of PrP-GPI transfer from solution to the cell membrane, the cationic liposome-based transfection agent Pro-Ject was employed (26). For quantification and statistical interpretation of colocalization between PrP-GPI and the cellular membrane, Pearson's (r_p_ or PCC) and Manders' (M_1_, M_2_) coefficients (27, 28) were calculated and evaluated with thresholds and significances (*p*-values) determined by the Costes' approach (29, 30) (Fig. 2*B*). Colocalization was proven with Manders' (M_1_, M_2_) coefficients close to 1 (Fig. 2*B*). In addition, a plot of the Van Steensel's cross-correlation function (CCF) (31) was obtained by shifting one of the SIM images relative to the other, which afforded a bell-shaped curve with its maxima located at δx = 0, confirming complete colocalization (Fig. 2*B*). The observed membrane-binding pattern agrees very well with findings reported for other PrP variants carrying a GPI anchor (mimic) and for PrP^C^ (20). On the contrary, PrP lacking a GPI anchor was not found to be located at the cellular membrane but occurred as small, bright spots that most likely constitute large PrP aggregates. Noteworthy, the GPI anchor mimic by itself showed a strong interaction with the cell membrane confirming its critical role in localizing PrP-GPI on the membrane (Fig. S2). Taken together, SR microscopy combined with quantitative and statistical colocalization analysis verified native-like membrane localization of our semisynthetic PrP-GPI species on SH-SY5Y cells.

### Impact of PrP-GPI on membrane organization

This was the basis for further studying the relation between PrP-GPI and the highly dynamic and spatially heterogeneous membrane on a molecular level by fluorescence correlation spectroscopy (FCS). Assessing diverse molecular dynamics in live specimen is a challenge well met by FCS. By measuring dynamic processes such as diffusion and interactions within typical timescales of micro- to milliseconds based on fluctuations of the fluorescence signal, FCS provides a quantitative approach to observe such mechanisms at the molecular level (32). For assessing PrP–membrane interactions, the membrane accumulation and mobility of Cy5-labeled PrP-GPI at the plasma membrane of SH-SY5Y cells were analyzed together with established membrane probes that are linked to different patterns of membrane organization. Treatments with methyl-β-cyclodextrin (mβCD), a cholesterol-depleting agent that changes membrane composition, and addition of latrunculin A (LatA), an actin polymerization inhibitor (33, 34, 35, 36), allow investigating the mobility of PrP-GPI within the membrane and can provide novel links to its function (37).

**Figure 3:**
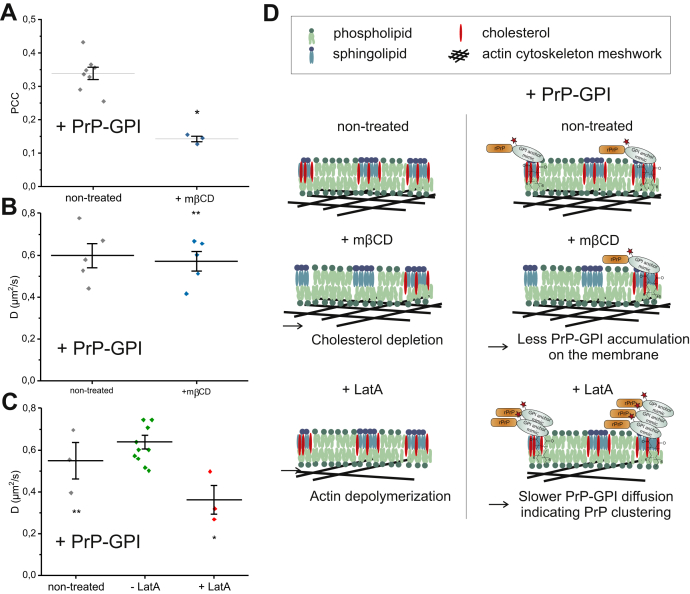
**FCS analysis of Cy5-labeled PrP-GPI on the cellular membrane by imaging and dynamic studies.***A*, Pearson's correlation coefficients (PCCs) for Cy5-labeled PrP-GPI and GFP-GPI AP on cells subjected to mβCD. Over a time period of 40 min, PrP–GPI accumulation was analyzed for mβCD-treated and nontreated cells. *B*, average D values for membrane-bound Cy5-labeled PrP-GPI on GFP-GPI AP transfected SH-SY5Y cells for non- and mβCD-treated cells. *C*, average D values for membrane-bound Cy5-labeled PrP-GPI on LifeAct-GFP transfected SH-SY5Y cells for non- and LatA-treated cells. PrP-GPI was measured before addition of LatA (-LatA) for 30 min and after LatA treatment (+LatA) for 20–75 min. Control measurements were conducted on nontreated LifeAct-GFP cells (nontreated) for the same period of time of PrP incubation (120 min). Mean values ± SD for all ACFs (3, 5, 11) are shown here that are identical to the number of measurements (N_m_) for confocal FCS. Statistical significance is indicated by ∗ (with *p* < 0.05 based on the two-sample *t*-test) and ∗∗ indicates no significant difference. *D*, schematic overview of mβCD and LatA effects on the membrane interaction of Cy5 labeled PrP-GPI.

To assess the role of cholesterol-dependent membrane domains on PrP-GPI membrane localization, SH-SY5Y cells transiently transfected with green fluorescent protein carrying a C-terminal glycosylphosphatidylinositol anchor (GFP-GPI AP) that acts as a cholesterol-dependent membrane domain marker were treated with mβCD, a cholesterol-depleting agent (38, 39, 40). The role of these cholesterol-rich domains in PrP-GPI plasma membrane anchoring was visualized by confocal imaging. Quantification was based on colocalization analysis of nontreated compared with mβCD pretreated GFP-GPI AP transfected SH-SY5Y cells incubated with Cy5-labeled PrP-GPI. Over a time period of 40 min, significantly less PrP-GPI accumulated at the cell membrane of mβCD pretreated cells (Fig. 3*A* and Fig. S3, Table S1). Thus, disturbances in the integrity of the cholesterol-rich, ordered membrane domains influence PrP–GPI membrane binding without affecting its diffusion properties (Fig. 3*B*). This finding agrees with other studies highlighting the necessity of cholesterol for PrP^C^ localization at the cell surface (8, 41, 42, 43).

Typically, the diffusion of membrane-anchored proteins such as GFP-GPI AP located in the ordered and tightly packed cholesterol-dependent domains is increased upon treatment with mβCD (38, 39), and a similar behavior was observed here (Table S2). Confocal FCS data of Cy5-labeled PrP-GPI on SH-SY5Y cell membranes was best fitted with a two-component model (2D, 2p) yielding the diffusion coefficients (D) and fractions of PrP-GPI in solution and bound to the cell membrane. The fast component represents PrP-GPI in the extracellular space not linked to the membrane (*D* ∼ 30 μm^2^/s, fraction 60%), and the slow component corresponds to membrane-bound PrP-GPI (*D* ∼ 0.6 μm^2^/s, fraction 40%), respectively. Here, we solely focus on the relevant slow component representing membrane-bound PrP-GPI.

The dynamics of Cy5-labeled membrane-bound PrP-GPI remained unaffected by mβCD treatment and retained a diffusion coefficient of ∼0.6 μm^2^/s (Fig. 3*B*, Table S3). This suggests a stronger anchoring of PrP-GPI within the cholesterol-dependent domains of the plasma membrane or the involvement of additional factors, *e.g.*, links to the cytoskeleton. Other studies of GPI-anchored proteins, such as the folate receptor and CD52, indicate a direct or indirect link to the actin cytoskeleton and binding of these GPI-anchored proteins to the plasma membrane (44, 45, 46). Such a link could explain the strong anchoring of PrP-GPI to the cellular membrane rendering its mobility unaffected by cholesterol depletion.

To address such an involvement of the cytoskeleton in PrP–GPI interactions on the membrane, we disrupted the cytoskeleton by exposing the cells to LatA (47). This treatment had no significant effect on the fraction of PrP-GPI not bound to the membrane (Fig. S4) and also did not render membrane-bound PrP-GPI more mobile as could be expected based on previous observations with proteins carrying a transmembrane domain such as the epidermal growth factor receptor (EGFR). Bag *et al.* showed an increase in both the fractions of slow-diffusing and fast-diffusing EGFR populations after LatA treatment that were attributed to synergistic effects of EGFR clustering on removal of the cytoskeleton barrier and a release of cytoskeleton-bound EGFR, respectively (38, 48). In contrast, PrP–GPI cytoskeleton disruption caused a significant decrease in the diffusion coefficients from 0.6 ± 0.11 to 0.4 ± 0.12 μm^2^/s for membrane-bound PrP-GPI (Fig. 3*C*; Table S4; Fig. S5 shows confocal images of Cy5-labeled PrP-GPI bound to LifeAct-GFP transfected SH-SY5Y cells). Slower dynamics associated with clustering have been described previously for other peptides and proteins, including PrP itself (49, 50). Control measurements with GFP-GPI AP showed no effect of LatA treatment on mobility of this GPI-anchored membrane probe and exclude a general effect on GPI-anchored proteins (Fig. S6, Table S5).

## Discussion

Elucidating the pathophysiologic events in prion diseases involves understanding the trigger(s) of the conformational change of cellular (PrP^C^) into scrapie prion protein (PrP^Sc^) that further propagates PrP^C^ misfolding and aggregation. To this end, recent studies describe fibril fragmentation and elongation of individual recombinant murine PrP aggregates from seeded aggregation in the test tube (51, 52). But so far, experimental studies have been very limited in directly analyzing the earliest events of PrP misfolding at the cellular membrane of live cells, the initial site of prion infection, by a lack of suitably modified PrP variants and sophisticated experimental setups. Here, application of a semisynthesis strategy offers a unique opportunity to access homogeneous membrane-anchored Cy5-labeled PrP (PrP-GPI) allowing to directly observe PrP on the cellular membrane. SR images confirmed a native-like membrane localization of Cy5-labeled PrP-GPI on SH-SY5Y cells. Upon manipulating the integrity of the cholesterol-rich, ordered membrane domains in the cell membrane, membrane binding of PrP-GPI decreased but the diffusion dynamics of membrane-bound PrP-GPI remained unaffected. Cytoskeleton disruption, however, caused slower diffusion of PrP-GPI on the membrane. We propose that these slower dynamics of Cy5-labeled PrP-GPI at the membrane are linked to PrP clustering and constitute the initial step of PrP aggregation. Providing direct evidence for PrP clustering on live cells is quite challenging. For example, Goold *et al.* (8) have previously analyzed a PrP knockdown cell line expressing epitope-tagged PrP^C^ upon infection with exogenous PrP^Sc^ by immunostaining. Shortly after prion exposure, PrP^Sc^ was detected on the plasma membrane by immunostaining. This is, however, only feasible on fixed cells and impedes dynamic studies. According to the picket-fence model by Fujiwara *et al.* (34, 35, 36), the plasma membrane consists of membrane compartments formed due to partitioning of the entire plasma membrane by the actin cytoskeleton (fence) and transmembrane proteins anchored to the cytoskeleton (pickets, Fig. 3*D*). The hydrodynamic friction of the immobilized transmembrane proteins causes the diffusion of membrane components around them to slow down. Thus the physical and diffusion barriers provided by the cytoskeleton and the immobilized transmembrane proteins provide an explanation for plasma membrane molecules undergoing short-range free diffusion within a fence with a diffusivity similar to free diffusion in model membranes, as described by the fluid mosaic model (53), but also slower long-range diffusion across multiple fences. By inhibiting actin polymerization, a physical and diffusion barrier provided by the cytoskeleton was removed, which enabled PrP–GPI clustering and aggregation on the cellular membrane as an early step in PrP^C^ conversion (Fig. 3*D*). Our findings suggest that an intact actin cytoskeleton can act as a barrier for the conversion of PrP^C^ into PrP^Sc^ on cell membranes. They open new avenues for understanding the molecular basis of the early steps of PrP aggregation as well as routes to interfere with these steps to prevent prion protein-based diseases.

## Experimental procedures

### Protein expression and purification

The PrP-α-thioester (aa 23–231) was obtained according to previously established procedures in the Becker group (20, 22, 54). Briefly, a pTWIN1 plasmid containing a construct of PrP fused to *Mxe* GyrA intein and a chitin-binding domain (CBD) was transformed into *E. coli* chemical-competent Rosetta 2 cells (Fig. S7). After expression in inclusion bodies, the fusion construct was solubilized and purified under denaturing conditions using immobilized metal affinity chromatography (IMAC). MESNA-mediated intein cleavage and concomitant thioester formation proceeded in 4 M urea buffer (pH 8) supplemented with 500 mM sodium 2-mercaptoethanesulfonate (MESNA) (Fig. S8). The crude reaction mixture was purified *via* preparative reversed-phase high-performance liquid chromatography (RP-HPLC). Desired fractions were identified *via* electrospray ionization–mass spectrometry (ESI-MS) operating in positive ion mode, combined and following to lyophilization stored at –80 °C. Purity of the isolated α-thioester was assessed *via* analytical RP-HPLC (Fig. S9).

### Folding of Cy5-labeled PrP variant

Folding of the Cy5-labeled PrP variant equipped with a GPI anchor mimic into its native structure was accomplished according to a protocol from Chu *et al.* (22). Unless otherwise stated, all steps were performed at 4 °C. A solution of denatured modified PrP in 6 M Gdn·HCl, 50 mM Tris-HCl buffer (pH 8) was stepwise diluted to 2.5 M Gdn·HCl, using 20 mM sodium acetate buffer (pH 5), containing a redox system of 0.3 and 3 mM oxidized and reduced glutathione (GSSG, GSH), and additionally 20 mM *N*-octyl-*β*-*D*-glucopyranoside (OG). After the folding reaction, PrP was dialyzed against 20 mM sodium acetate buffer (pH 5), with 20 mM OG, respectively, using Slide-A-Lyzer cassettes with MWCO of 10 kDa. Precipitates of misfolded protein were separated *via* centrifugation at 14,000*g* for 15 min. Supernatants containing the folded PrP variant were stored at –80 °C.

### SDS–polyacrylamide gel electrophoresis (SDS-PAGE)

Protein analysis was carried out by sodium dodecyl sulphate–polyacrylamide gel electrophoresis (SDS-PAGE) based on a protocol from Laemmli *et al.* (55) using 15% acrylamide gels under reducing conditions. Samples were treated in 1:1 (v/v) ratio with SDS-loading buffer (pH 6.8), containing 0.5 M Tris-HCl, 6% (w/v) SDS, 35% (w/v) glycerol, 3.5% (v/v) β-mercaptoethanol, 0.05% (w/v) bromophenol blue, boiled at 95 °C, and loaded onto the stacking gel. Prior to that any salts in higher concentrations had been removed *via* precipitation with 40% (v/v) trichloroacetic acid (TCA). Applied voltage on power source from VWR was set to 250 V for 35 min. Gels were stained with Coomassie Brilliant Blue R-250 or silver (56). Detection was performed on ChemiDoc MP Imaging System from Bio-Rad with Image Lab 5.1 software.

### Solid-phase peptide synthesis (SPPS)

SPPS was performed applying Fmoc-strategy on a preloaded Fmoc-A-Wang resin, in scales of 0.03–0.25 mmol to afford a peptide based on the sequence H – CKGENLYFQSKAAKK-PPO_3_-A – OH, according to a protocol from Olschewski *et al.* (20). Coupled amino acids carried orthogonal side-chain protecting groups as follows: Lys(Boc), Lys(Mtt) and Lys(ivDde), respectively, Ala, Ser(tBu), Gln(Trt), Phe, Tyr(tBu), Leu, Asn(Trt), Glu(OtBu), Gly, and Boc protected Cys(Trt). Synthesis was accomplished either manually in syringes and glass frits (pore size 2) or automatically using CEM Liberty Blue, Automated Microwave Peptide Synthesizer (CEM). For deprotection of Fmoc, a solution of 20% (v/v) piperidine in *N*,*N*-dimethylformamide (DMF) was used in cycles of 3 and 7 min. All amino acids were coupled for 30 min in amounts of 2.5eq and 4.8eq, respectively, using either 2.38eq 0.5 M (2-(1*H*-benzotriazol-1-yl)-1,1,3,3-tetramethyluronium hexafluorophosphate (HBTU) in DMF and 5eq *N*,*N*-diisopropylethylamine (DIEA) or 4.5eq 0.5 M *N*,*N*′-diisopropylcarbodiimide (DIC) and 5eq 1 M Oxyma in DMF. To enhance solubility, polyethyleneglycol polyamide oligomer (PPO) as trimer was introduced, using first 10eq succinic anhydride in 5eq 0.5 M 1-hydroxybenzotriazole (HOBt) in DMF and 6eq DIEA, second 20eq 0.5 M 1,1′-carbonyldiimidazole (CDI) in DMF, and last 12eq of each, 0.5 M HOBt and 4,7,10-trioxatridecane-1,13-diamine. All steps were conducted for 30 min. For lipidation, ε-amino groups of lysine 11 and 14 were palmitoylated. After selective removal of orthogonal methyltrityl (Mtt) protective groups with 1% (v/v) trifluoroacetic acid (TFA), 1% (v/v) triisopropylsilane (TIS) in dichloromethane (DCM), palmitoylation was carried out with 20eq palmitoyl chloride, 20eq HOBt, and 22eq triethylamine in DCM:DMF (3:1) solution for 12 h. Coupling of the cyanine 5 (Cy5) dye to ε-amino group of lysine 2 was performed over 1 h with *in situ* activation using 1.5eq Cy5 with 1.44eq 0.5 M HBTU in DMF and 3eq DIEA. Cy5 synthesis was accomplished in three steps, according to protocols from Yamane *et al.*, Zhang *et al.* and Korbel *et al.* (57, 58, 59) (Fig. S10–13, Scheme S1). At the end of SPPS, the peptide was cleaved off from resin through incubation with a solution of 92.5% (v/v) TFA, 5% (v/v) TIS, 2.5% (v/v) dd H_2_O for 2.5 h. The crude peptide was obtained through precipitation with cooled diethyl ether, subsequently dissolving in acetonitrile:dd H_2_O (1:1), 0.1% (v/v) TFA and lyophilization. For purification, semi- and preparative RP-HPLC was conducted. The crude peptide was dissolved in 6 M Gdn·HCl buffer (pH 4.7). Desired fractions were identified *via* ESI-MS operating in positive ion mode, combined and following to lyophilization stored at –20 °C (Table S6). Purity of the Cy5-labeled peptide was assessed *via* analytical RP-HPLC (Fig. S14).

### Expressed protein ligation (EPL)

The ligation between PrP α-thioester (aa 23–231) and the Cy5-labeled, lipidated peptide proceeded under argon at 37 °C and shaking at 600 rpm. A 2.5-fold excess of the peptide was subjected to 1 mM of PrP α-thioester. The two components reacted in degassed buffers (pH 7.2) consisting of 6 M Gdn·HCl, 0.2 M Na_2_HPO_4_, with 20 mM TCEP and as thiol catalyst 30 mM 4-mercaptophenylacetic acid (MPAA) for 6 h. Progress was monitored by analytical RP-HPLC (Fig. S15, Table S7). The reaction was complete as judged by time-dependent HPLC traces revealing consistent integrated peak areas of the ligation product and LC/MS spectra exposing complete hydrolysis of the thioester. Subsequently, the reaction mixture was diluted with acidic 6 M Gdn·HCl buffer (pH 4.7) and subjected to RP-HPLC. Desired fractions were identified *via* ESI-MS operating in positive ion mode, combined and following to lyophilization stored at –80 °C. Purity of Cy5-labeled PrP variant containing a GPI anchor mimic was assessed *via* analytical RP-HPLC (Figs. S16 and S17).

### HPLC and mass spectrometry

RP-HPLC was conducted on a Waters Auto Purification HPLC/MS system (3100 Mass Detector, 2545 Binary Gradient Module, 2767 Sample Manager, 2489 UV/Visible Detector) and a Varian ProStar HPLC system, respectively. On a semi- and preparative scale, separation was achieved using Kromasil (300–10-C4, 250 × 21.2 mm and 250 × 10 mm, 10 μm particle size) and Grace Vydac C4 columns (250 × 22 mm and 250 × 10 mm, 5 μm particle size) running linear gradients of 30–60% for PrP α-thioester, 30–90% for peptide, and lipidated PrP variant of buffer B (acetonitrile +0.05% TFA) in buffer A (dd H_2_O + 0.05% TFA) over 60 min. Analytical LC/MS was performed using a Kromasil C4 column (300-5-C4, 50 × 4.6 mm, 5 μm particle size) at a flow rate of 1 ml/min running linear gradients of 5–65% or 5–95% over 10 min with buffer compositions and ascribed compounds as mentioned above. Analytical RP-HPLC was conducted on a Dionex Ultimate 3000 instrument using a Kromasil C4 column (300-5-C4, 150 × 4.6 mm, 5 μm particle size) at a flow rate of 1 ml/min running linear gradients of 5–65% for the PrP α-thioester, 5–95% for the peptide, and lipidated PrP variant of buffer B (acetonitrile +0.08% TFA) in buffer A (dd H_2_O + 0.1% TFA) over 30 and 40 min, respectively. As required, HPLC traces were monitored at absorptions of 214 and 280 nm and at emission of 650 nm. The injection peak present in 0–5 min of the HPLC runs was cut off in the depicted chromatograms. HPLC traces of the ligation time courses show only time periods comprising the peaks used for integration. Mass spectra from Waters Auto Purification HPLC/MS system were acquired by electrospray ionisation (ESI) operating in positive ion mode. Deconvolution was accomplished using the softwares MassLynx and MagTran. All raw data were exported and processed using OriginPro.

### Circular dichroism (CD)

CD spectra were recorded on a Chirascan Plus CD-spectrophotometer from Applied Photophysics using a micro cuvette with 1 mm path length from Hellma Analytics. Each spectrum was acquired at 25 °C from 190 to 260 nm in 1 nm steps. In total, ten measurements were averaged, and the background was substracted. The raw data was exported from Pro-Data software and further processed using OriginPro. Spectra were analyzed regarding their secondary structure by the curve-fitting software CDNN from Böhm *et al.* (60). For each measurement, a final protein concentration of 4–10 μM was used that previously had been determined by NanoDrop 2000c from Fisher-Scientific. For folded recombinant, lipidated and labeled PrP variants, respectively, calculated molar extinction coefficients at 280 nm obtained from Expasy-ProtParam (https://web.expasy.org/protparam) were used in combination with MW values as follows: recombinant PrP (aa 23–231) 63,495 M^−1^ cm^−1^, 23,200.6; lipidated PrP 64,985 M^−1^ cm^−1^, 26,352.6 Da, and Cy5-labeled PrP 26,803.9 Da.

### Cell culture, staining, and treatment

SH-SY5Y and stably transfected LifeAct-GFP SH-SY5Y cells were cultivated in DMEM medium supplemented with 10% FBS and 1% PS at 37 °C in 5% (v/v) CO_2_ humidified environment. For the staining of the cell membrane using CellMask green, cells were incubated with dilutions in live cell imaging solution of 1:1000 for 30 min at 37 °C in 5% (v/v) CO_2_ humidified environment. Transfection of GFP-GPI AP by electroporation into live SH-SY5Y cells was accomplished on a Neon Transfection System from Life Technologies. Approxmately 90% confluent cells grown in a 25 cm^2^ culture flask were washed with 1× PBS and collected after trypsinization with 1× Trypsin solution *via* centrifugation for 3 min at 3000 rpm using a 5810 centrifuge from Eppendorf. Pelleted cells were resuspended in 12.5 μl of R-buffer, mixed with ∼1 μg of plasmid per 8× well chamber, drawn into a 10 μl Neon transfection tip, and electroporated at 1200 V for 20 ms with three pulses in a Neon transfection tube containing 3 ml of E-buffer. After transfection, cells were plated and grown in culture medium at 37 °C in 5% (v/v) CO_2_ humidified environment for 48 h. SH-SY5Y cells were washed twice with 1× Hanks' Balanced Salt Solution (HBSS) before mounting them into the microscope chamber containing 1× HBSS as imaging medium. For drug treatments to inhibit actin polymerization and deplete cholesterol, respectively, the imaging medium was replaced with 3 μM LatA diluted from a 100 μM stock solution in DMSO and 3 mM mβCD, both dissolved in HBSS. Folded PrP variant in sodium acetate buffer containing OG (pH 5) was added to the cells 10× diluted in HBSS to a final concentration of 200 nM. For encapsulation of PrP by Pro-Ject, PrP was treated according to the manufacturer's protocol. For nonlive measurements, cells were post fixed with formaldehyde (3.7% in PBS, 20 min) and mounted adding Roti-Mount FluorCare.

### Structured illumination microscopy (SIM) imaging and colocalization analysis

SR imaging using structured illumination microscopy (SIM) technique was conducted on a Zeiss 710 confocal laser scanning microscope (CLSM) with an Elyra PS.1 system at 37 °C. The 488 nm (for CellMask green excitation) or the 642 nm (for Cy5 excitation) HR-diode laser beam was focused on the sample by an oil immersion objective (Plan-Apochromat DIC M27, 63×, NA1.4). Fluorescence signal from the sample was spectrally filtered *via* an emission filter, namely BP495-575/LP750 for CellMask green or LP655 for Cy5 emission and recorded by an electron multiplying charge-coupled device (EMCCD) camera Andor iXon 897. Images with a field of view of 1280 × 1280 pixels and pixel sizes of 64 × 64 × 110 nm were acquired in five phases and rotations, using grating periods of 28 and 34 μm for CellMask green and Cy5, respectively. Postmeasurement image reconstruction was accomplished using Zen 2012 SP3 (black) software from Zeiss with the theoretical point spread function (PSF) set to a value of 2, a baseline cut, the SR frequency weighting set to 1, the noise filter (NF) to 4 (for Cy5) and 3 (for CellMask green), and sectioning at 83/83/100. Eventually structured illumination images with a field of view of 2430 × 2430 pixels and pixel sizes of 32 × 32 × 110 nm were obtained. Lateral resolution determined by Zen is 118 nm for CellMask and 129 nm for Cy5 and determination with ImageJ gives values of 122 nm for CellMask and 124 nm for Cy5. For final image representation, SIM images were displayed using 3D shadow rendering and adjusted with regard to their brightness and contrast. For quantitative colocalization analysis, regions of interest (ROIs) with 753 × 313 pixels were cut out.

Thereby, precise local information of PrP within cells could be extracted from the image stacks using ImageJ (61, 62) software with Just Another Colocalization Plugin (JACoP) (30, 63). Several global statistic approaches performing intensity correlation coefficient-based (ICCB) analyses that offer complementary information were applied and compared. Pearson's correlation coefficient (PCC) describes the spread of the pixel distribution within a scatter plot in respect to the fitted line displaying the relationship between the intensities of the two images. Its value lies in the range from 1 to –1, with 1 for complete colocalization and –1 for exclusion (Equation 1).

*Equation 1. Pearson's correlation coefficient (PCC)* with A_i_ and B_i_ as the intensities of channel A and B, a and b as the corresponding mean intensities.

(1) $$r_{p}\;=\;\frac{\sum_{i}\left(A_{i}\;-\;a\right)\cdot \left(B_{i}\;-\;b\right)}{\sqrt{\sum_{i}{\left(A_{i}\;-\;a\right)}^{2}\cdot \sum_{i}{\left(B_{i}\;-\;b\right)}^{2}}}$$

To provide statistical significance to the calculated PCCs, Van Steensel's (31) and Costes' (29) approachs were conducted. For the Van Steensel's cross-correlation function (CCF), the PCC is calculated while a shift of one of the images relative to the other is operated. If the resulting plot of the PCC as a function of the displacement shows a bell-shaped curve or a dip, colocalization or exclusion is identified. Depending on the position of the maximum being at 0 or shifted, partial colocalization can be determined. The height correlates with noise and/or differing fluorescence intensities. Costes' approach offers two possibilities, namely automatic thresholding together with a calculation of the respective PCC and by comparing a randomized with the original image an evaluation of the significance (*p*-value) of the PCC, meaning the probability of obtaining the specified PCC by chance. In Costes' automatic thresholding, initial limits are set to the maximum intensity of each channel and progressively decremented. PCCs are calculated for each increment of thresholded image pairs. The final Costes' thresholds are then set to values below which PCC is zero or negative. Another ICCB analysis is provided by Manders' coefficients (27, 28). These values are built by the ratio of the sum of intensities of colocalizing pixels from one channel and its integrated density. Pixels from image A are considered colocalized, if their intensity in channel B is above 0 (M_1_), and vice versa (M_2_). Values range from 0 to 1 for non- and complete overlapping events (Equation 2).

*Equation 2. Manders' coefficients* with A_i,coloc_ = A_i_, if B_i_ > 0 and B_i,coloc_ = B_i_, if A_i_ > 0.

(2) $$\begin{array}{l} M_{1}=\frac{\sum_{i}A_{i,coloc}}{\sum_{i}A_{i}}\\ M_{2}=\frac{\sum_{i}B_{i,coloc}}{\sum_{i}B_{i}} \end{array}$$

An interpretable representation of colocalization was described by Li *et al.* (64) (see Figs. S18 and S19). A set of two graphs presents the intensity correlation analysis (ICA) results. Normalized intensities (0–1) are plotted as a function of the product (A_i_ − a)(B_i_ − b) for each channel, with the upper-case characters as the current pixel's intensity and the lower-case characters as the channel's mean intensity. For colocalizing pixels, the covariance of both channels is positive, resulting in a dot cloud concentrated on the right side of x = 0. Non-colocalizing pixels are located on the left side. In case of ambiguous results, the intensity correlation quotient (ICQ) can be determined. It is defined as the ratio of positive (A_i_ − a)(B_i_ − b) products divided by the overall products subtracted by 0.5. Thus, resulting ICQs vary from colocalization with 0.5 to exclusion with –0.5, whereas random staining and images corrupted by noise give values close to 0.

### Confocal imaging and fluorescence correlation spectroscopy (FCS)

Both confocal imaging and FCS were conducted on an Olympus FV1200 laser scanning confocal microscope from Olympus IX83 equipped with a Microtime 200 upgrade kit for single molecule detection from PicoQuant at 37 °C. The 488 nm argon-ion (for Atto488 and EGFP excitation) or the 635 nm (for Atto655 and Cy5 excitation) laser beam from Melles Griot was focused on the sample by a water immersion objective (UPLSAPO, 60×, NA1.2) from Olympus after being reflected by a dichroic mirror (DM405/488/543/635 bandpass) from Olympus and a scanning mirror unit. The laser powers measured before the objective were 17 and 12 μW for the 488 nm and the 635 nm lasers, which correspond to excitation intensities of 17 and 8 kW/cm^2^, respectively (65).

For confocal imaging, the system was operated in scan mode. Fluorescence signal from the sample was passed through the same objective, through a 120 μm pinhole in the image plane to block off out-of-focus light, spectrally filtered *via* a bandpass emission filter, namely BA505-525 for EGFP or BA655 to 755 for Cy5 excitation, from Olympus, and recorded by a photomultiplier detector. Images with a field of view of 512 × 512 pixels and a pixel size of 138 nm were acquired at a scan rate of 12.5 μs/pixel.

For confocal FCS measurements, the system was descanned and operated in point mode. Fluorescence signal from the sample was passed through the 120 μm pinhole, spectrally filtered by an emission filter, namely 513/17 (for Atto488 or EGFP emission) or 680/42 (for Atto655 or Cy5 emission) from Semrock, and eventually recorded by a sensitive single-photon avalanche photodiode (SPAD) SPCEM-AQR-14 from PerkinElmer, Optoelectronics. Signal detected by the avalanche photodiode was then processed online by the SymPhoTime 64 software correlator from PicoQuant, giving the autocorrelation function (ACF). Postmeasurement fitting was performed in the SymPhoTime 64 software or in Igor Pro 7 from WaveMetrics using the open-source FCS 2.1 script provided by the Wohland laboratory at NUS (available at: http://www.dbs.nus.edu.sg/lab/BFL/confocal_FCS.html). The total measurement time for the calibration dyes and on the cells was in the range of 30–40 s.

Prior to conducting FCS measurements on the samples, the system was aligned for performance of the highest SNR achievable by adjusting the pinhole position in x-, y-, and z-directions. To obtain the maximum fluorescence intensity emitted by the respective calibration dyes, an FCS measurement was conducted to generate an ACF, which was then fitted to obtain the structure factor *K* (Equation 4) and the molecular brightness (*η*) in photon counts per particle per second (cps). The pinhole position was adjusted and FCS measurements repeated until a *K* value of 4–6 and the maximum *η* were achieved. As the water immersion objective used had a cover slip correction collar, this was also adjusted to optimize the brightness. Using the diffusion time of the calibration dye ($\tau_{D,calibrationdye}$) with known diffusion coefficient and *K* values extracted from the FCS fits, the effective observation volume was then calculated applying Equations (3), (4), (5).

*Equation 3. Effective observation volume* (*V*_*eff*_) in confocal FCS for noninteracting particles, with $\vec{r}$ as the spatial coordinate and $MDE\left(\vec{r}\right)$ the molecular detection efficiency.

(3) $$V_{eff}=\frac{{\left({∭}_{-\infty }^{\infty }MDE\left(\vec{r}\right)dV\right)}^{2}}{{∭}_{-\infty }^{\infty }{\left(MDE\left(\vec{r}\right)\right)}^{2}dV}=\pi^{\frac{3}{2}}\omega_{0}^{2}z_{0}$$

*Equation 4. Structure factor K* is defined as the ratio of the axial (*z*_0_) and radial (*ω*_0_) distances of the laser focal spot at 1/e^2^ value of the maximum intensity at the focus of the observation volume.

(4) $$K=\frac{z_{0}}{\omega_{0}}$$

*Equation 5. Diffusion time and coefficient*.

(5) $$\tau_{D}=\frac{\omega_{0}^{2}}{4D}$$

The appropriate ACF fitting models are chosen considering the weight of the noise from each data point, *i.e.*, the standard deviation as described by Koppel (66) and Wohland *et al.* (67). Theoretical models of the ACF *G*(*τ*) can be described considering the average number of particles within the observation volume (*N*), the lag time (*τ*), the diffusion time (*τ*_*D*_), the structure factor *K*, and the convergence value of the ACF at long lag times (*G*_∞_) with an expected value of . In the case of multiple diffusion components, *G*(*τ*) is expressed as the linear sums of the individual components weighted with their respective mole fractions (*F*_*i*_) of the *i*^*th*^ species. For triplet-state photodynamics, *F*_*trip*_ refers to the fraction and *τ*_*trip*_ to the relaxation time of the triplet state (68, 69). For the calibration dye solutions, 3D diffusion fitting models with a single component (1p), 3D,1p, or with an additional single triplet contribution (1t), 3D,1p1t, were used (Equation 6). For the membrane measurements, the fitting models used were 2D diffusion with two diffusion components (2p), 2D,2p, or with an additional single triplet contribution, 2D,2p1t (Equation 7). As the photophysical processes of Cy5 are strongly environment sensitive, but not believed to change the diffusion properties drastically, they were neglected during the fitting of the ACF curves and a 2D, 2p model was applied on data from later lag times (70).

*Equation 6. Mathematical models for 3D diffusion of a single component (1p)* without (upper formula) and with a single triplet contribution (lower formula) for confocal FCS.

(6) $$\begin{array}{l} G_{3D,1p}\left(\tau \right)\;=\;\frac{1}{N}{\left(1+\frac{\tau }{\tau_{D}}\right)}^{-1}{\left(1+\frac{\tau }{K^{2}\tau_{D}}\right)}^{-1/2}+\;G_{\infty }\\ G_{3D,1p1t}\left(\tau \right)=\frac{1}{N}{\left(1+\frac{\tau }{\tau_{D}}\right)}^{-1}{\left(1+\frac{\tau }{K^{2}\tau_{D}}\right)}^{-1/2}\left(1+\left(\frac{F_{trip}}{1-F_{trip}}\right)e^{-\frac{\tau }{\tau_{trip}}}\right)+G_{\infty } \end{array}$$

*Equation 7. Mathematical models for 2D diffusion with two components (2p)* without (upper formula) and with a single triplet contribution (lower formula) for confocal FCS.

(7) $$\begin{array}{l} G_{2D,2p}\left(\tau \right)=\frac{1}{N}\left(\left(1-F_{2}\right){\left(1+\frac{\tau }{\tau_{D1}}\right)}^{-1}+F_{2}{\left(1+\frac{\tau }{\tau_{D2}}\right)}^{-1}\right)+G_{\infty }\\ G_{2D,2p1t}\left(\tau \right)=\frac{1}{N}\left(\left(1-F_{2}\right){\left(1+\frac{\tau }{\tau_{D1}}\right)}^{-1}+F_{2}{\left(1+\frac{\tau }{\tau_{D2}}\right)}^{-1}\right)\left(1+\left(\frac{F_{trip}}{1-F_{trip}}\right)e^{-\frac{\tau }{\tau_{trip}}}\right)+G_{\infty } \end{array}$$

The fit parameters are the average number of particles *N*, diffusion time *τ*_*D*_, triplet relaxation time *τ*_*trip*_ and *G*_∞_. The *K* values obtained from the calibration dye solutions were fixed for all sample measurements (Table 1). The apparent *D* of the sample ($D_{sample}$) was determined using the known *D* of the calibration dye ($D_{calibrationdye}$), namely 400 (71) and 426 μm^2^/s (72, 73) for Atto488 and Atto655, respectively, together with the measured diffusion times of the sample and the calibration dye deriving from the fits of the ACF curve (Equation 8).

**Table 1:** Typical values of parameters fitted with 3D,1p and 3D,1p1t models for FCS calibration using 2 nM Atto488 and 3.8 nM Atto655 dye in PBS (pH 7.4)

Fit parameters	Fitted values	
	Atto488	Atto655
*N*	0.48 ± 0.000	0.85 ± 0.021
$\tau_{D}$ (μs)	22.6 ± 0.51	26.5 ± 0.13
$\tau_{trip}$ (μs)	3.5 ± 0.72	-
$F_{trip}$	0.2 ± 0.02	-
*K*	4.2 ± 0.12	4.5 ± 0.54
*G*_∞_	0.00036 ± 0.000243	−0.00009 ± 0.000748

The fitted values are given as value ± error of fit.

*Equation 8. Relationship between diffusion times and coefficients of the sample and calibration dye*.

(8) $$D_{sample}=\frac{\tau_{D,\;calibration\;dye}\cdot D_{calibration\;dye}}{\tau_{D,sample}}$$

In our studies, FCS measurements were conducted at *z*-positions below the upper membrane at the cell boundary. Cells were incubated with PrP and Project for 30 min, washed with HBSS buffer, and then subjected to treatments with methyl-β-cyclodextrin (mβCD) or latrunculin A (LatA) in concentrations of 3 mM and 3 μM, respectively. It should be noted that measurements at the upper membrane were occasionally hampered by PrP in solution that freely diffused and showed a stronger tendency to aggregate, resulting in a large number of spikes present in the fluorescence intensity traces complicating ACF analysis. This was rectified by segmenting the intensity trace in the absence of large fluorescence spikes and computing the ACFs thereafter.

### Imaging total internal reflection–FCS (ITIR-FCS)

Quantitative images of FCS parameters were obtained applying ITIR-FCS on an IX2-RFAEVA-2 total internal reflection fluorescence module on an IX71 microscope (Olympus) equipped with a high NA oil-immersion objective (PlanApo, 100×, 1.45, Olympus) and a back-illuminated EMCCD camera (Andor iXon 860, 128 × 128 pixels) at 37 °C in 5% (v/v) CO_2_ humidified environment. The system was optimized as described previously (74, 75). The air-cooled 488 nm argon-ion (for EGFP excitation) laser beam from Spectra-Physics Lasers was focused on the sample after being reflected by a combination of tilting mirrors and a dichroic mirror (495LP). TIR was achieved by adjusting the same combination of tilting mirrors to control the incident angle of the laser beam. A stack of 50,000 images was collected by the CCD chip with 2 ms time resolution. Andor Solis was used as the acquisition software and operated in kinetic mode. The fluorescence intensity fluctuations at each pixel of a ROI with a typical size of 5.04 × 5.04 μm^2^ containing 21 × 21 pixels on the sample were then bleach-corrected with a fourth-order polynomial, processed to yield ACFs and fitted according to Equation 9 for a one particle diffusion model by ImageJ (61, 62) software using the ImFCS 1.52 (76) plugin to produce quantitative maps of the diffusion coefficients (D) and the number of particles (N).

*Equation 9*. ITIR-FCS fitting model for 2D diffusion with one component (1p).

(9) $$G\left(\tau \right)\;=\;\frac{1}{N}{\left[erf\left(p\left(\tau \right)\;+\;\frac{1}{p\left(\tau \right)\sqrt{\pi }}\left(e^{-{\left(p\left(\tau \right)\right)}^{2}-1}\right)\right)\right]}^{2}+G_{\infty };\;p\left(\tau \right)\;=\;\frac{a}{2\sqrt{D_{\tau }\;+\;\sigma^{2}}}$$

In the above equation, G(τ) is the ACF as a function of the correlation time (τ) and N, *a*, D, and σ are the number of particles per pixel, pixel side length, diffusion coefficient, and standard deviation of the Gaussian approximation of the microscope PSF, respectively. The pixel size *a* of the CCD chip in the camera is 24 μm, which corresponds to 240 nm in the sample plane. G_∞_ is the convergence value of the ACF at very long correlation time. N, D, and G_∞_ were used as fit parameters.

## Data availability

We have included all data on protein synthesis and characterization in this article and the accompanying supporting information. Microscopy data analyzed for the article is summarized in figures and tables in here and in the supporting information. Additional images and FCS data sets are available upon request from Christian Becker, Institute of Biological Chemistry, University of Vienna, Austria (christian.becker@univie.ac.at), or Thorsten Wohland, Departments of Biological Sciences and Chemistry and Centre for Bioimaging Sciences (CBIS), National University of Singapore (NUS), Singapore (twohland@nus.edu.sg).

## Supporting information

This article contains supporting information (27, 28, 29, 31, 57, 58, 59, 64, 78, 79, 80).

## Conflict of interest

The authors declare that they have no conflicts of interest with the contents of this article.
