# Supplementary material for: Cytoskeleton-dependent clustering of membrane-bound prion protein on the cell surface
Source: J Biol Chem. 2021 Feb 2;296:100359. doi: 10.1016/j.jbc.2021.100359 (PMC7988330; doi:10.1016/j.jbc.2021.100359)
Supplement: Supplementary file 1 — Supporting information [file mmc1.pdf]

Supporting Information

Cytoskeleton-dependent clustering of membrane-bound prion protein on the cell surface

Stefanie Hackl, Xue Wen Ng, Danqin Lu, Thorsten Wohland and Christian FW Becker

Table of contents

Supporting Figures 4

Secondary structure of PrP 4

Figure S1. Far-UV CD spectra of PrP variants 4

Structured illumination microscopy (SIM) imaging & co-localization analysis 5

Figure S2. SR images & co-localization analysis of SH-SY5Y cells incubated with the Cy5 labeled GPI anchor-mimicking peptide 5

Confocal imaging & fluorescence correlation spectroscopy (FCS) 6

Figure S3. Cy5 labeled PrP-GPI incubated on GFP-GPI AP transfected SH-SY5Y cells non- and pre-treated with mβCD 6

Figure S4. Average D values for the non-membrane-bound Cy5-labeled PrP-GPI 7

Figure S5. Cy5 labeled PrP-GPI incubated on LifeAct-GFP transfected SH-SY5Y cells 7

Figure S6. ITIR-FCS results of GFP fluorescence from GFP-GPI AP subjected to LatA 8

Protein expression and purification 9

Figure S7. Following expression of PrP-Mxe GyrA intein-CBD fusion protein with SDS-PAGE analysis 9

Figure S8. MESNA-mediated intein cleavage reaction followed by SDS-PAGE 9

Figure S9. Characterization of PrP α-thioester 10

Synthesis of fluorophore Cy5 11

Scheme S1. Overview of Cy5 ***4*** synthesis 11

Figure S10. Characterization of Cy5 ***4*** 12

Figure S11. NMR spectrum of compound ***2*** 12

Figure S12. NMR spectrum of compound ***3*** 13

Figure S13. NMR spectrum of compound ***4*** 13

Figure S14. Characterization of Cy5 labeled GPI anchor-mimicking peptide..... 14

Expressed protein ligation (EPL)..... 15

Figure S15. Following the EPL reaction between the PrP-α-thioester and Cy5 labeled peptide by HPLC..... 15

Figure S16. Characterization of Cy5 labeled PrP-GPI *via* HPLC and MS 16

Figure S17. SDS-PAGE analysis of Cy5 labeled PrP-GPI 16

Co-localization analysis of super-resolution (SR) images..... 17

Figure S18. ICA plots of Cy5 labeled GPI anchor-mimicking peptide..... 17

Figure S19. ICA plots of Cy5 labeled PrP-GPI..... 17

Supporting Tables..... 18

Confocal imaging & fluorescence correlation spectroscopy (FCS) 18

Table S1. Pearson's correlation coefficients (PCCs) for Cy5 labeled PrP-GPI and GFP-GPI AP subjected to mβCD..... 18

Table S2. Confocal FCS results of the GFP fluorescence from GFP-GPI AP subjected to mβCD 18

Table S3. Confocal FCS results of the Cy5 fluorescence from PrP-GPI subjected to mβCD treatment..... 18

Table S4. Confocal FCS results of the Cy5 fluorescence from PrP-GPI subjected to LatA.... 19

Table S5. ITIR-FCS results of GFP fluorescence from GFP-GPI AP subjected to LatA 19

Solid phase peptide synthesis (SPPS)..... 19

Table S6. Yields of GPI anchor-mimicking peptide 19

Expressed protein ligation (EPL)..... 20

Table S7. Following the EPL reaction between the PrP-α-thioester and Cy5 labeled peptide by HPLC..... 20

Materials..... 21

Molecular biology 21

Chemicals.. 21

Cell culture.. 21

Supporting Figures

Secondary structure of PrP

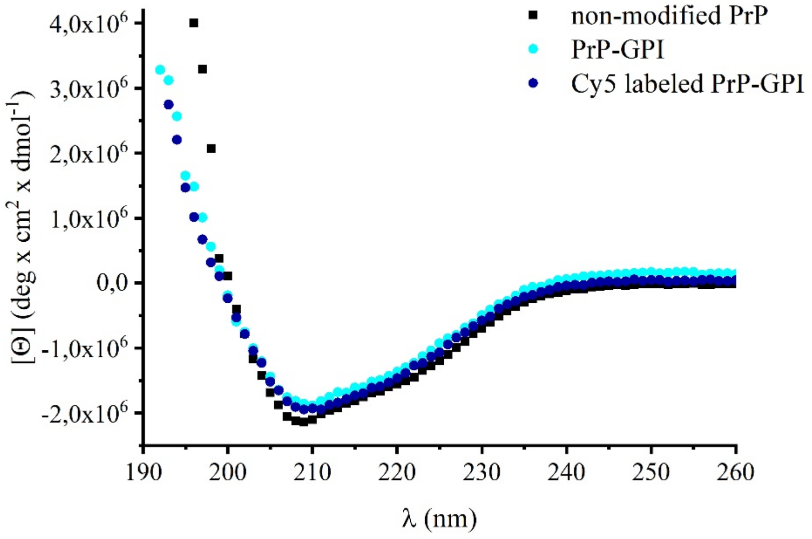

Figure S1. Far-UV CD spectra of PrP variants. The secondary structures of PrP-GPI and Cy5 labeled PrP-GPI are compared to their corresponding non-modified rPrP variant.

Structured illumination microscopy (SIM) imaging & co-localization analysis

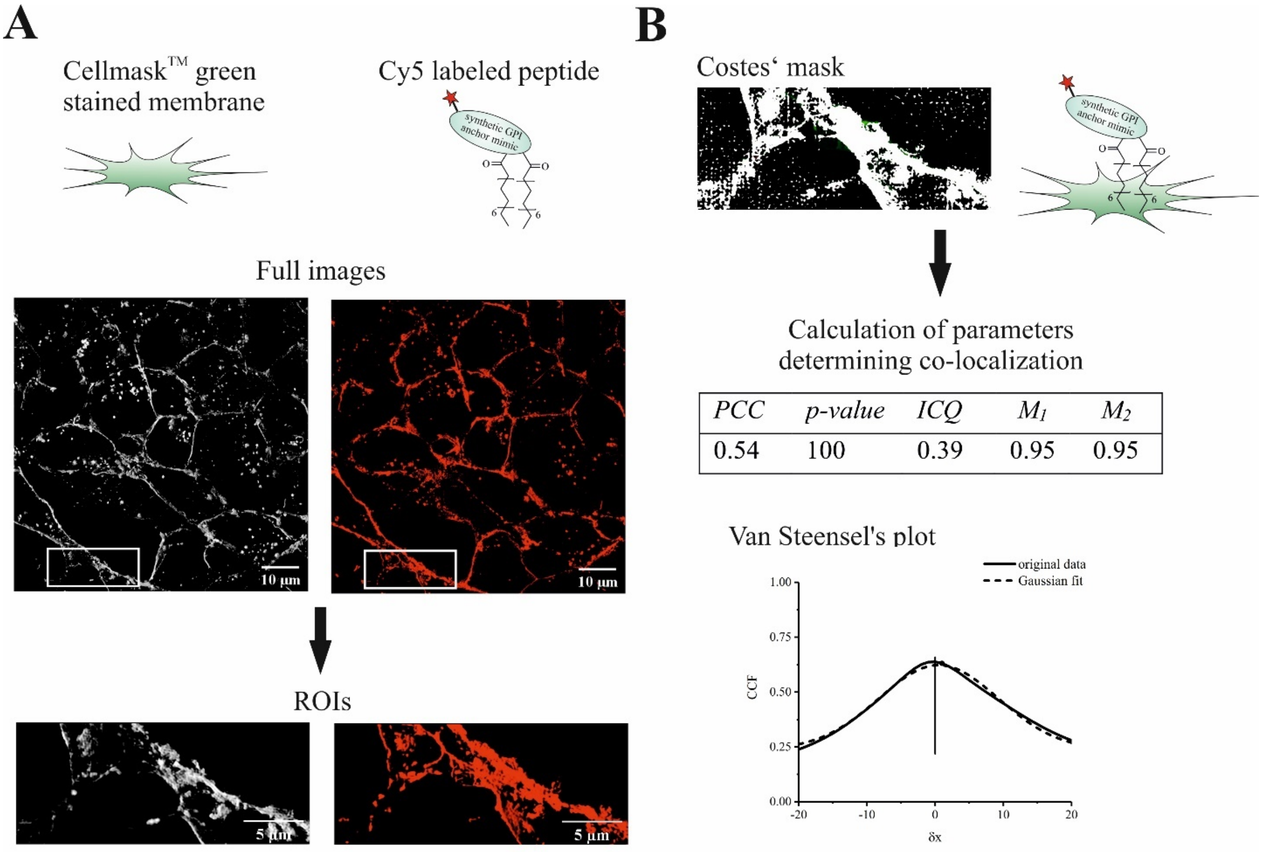

Figure S2. SR images & co-localization analysis of SH-SY5Y cells incubated with the Cy5 labeled GPI anchor-mimicking peptide. (**A**) SR images and the corresponding ROIs (regions of interest) used in the co-localization analysis of CellMask™ green (left) and Cy5 fluorescence (right) of the membrane and PrP. (**B**) A maximum intensity Z-projection of the Costes' mask (white: co-localization, black: background, red: Cy5 fluorescence, green: CellMask™ green fluorescence) illustrates the co-localization based on calculated thresholds according to Costes' statistical significance algorithm (29). Based on that, the co-localization was quantified with calculated intensity correlation quotients (ICQ) (64), Pearson's (PCC) and Manders' coefficients (M_1, M_2) (6,7), and statistically evaluated with *p*-values by Costes' (29). Additional evidence for co-localization was provided by the plot of the Van Steensel's cross-correlation functions (CCFs) (31).

Confocal imaging & fluorescence correlation spectroscopy (FCS)

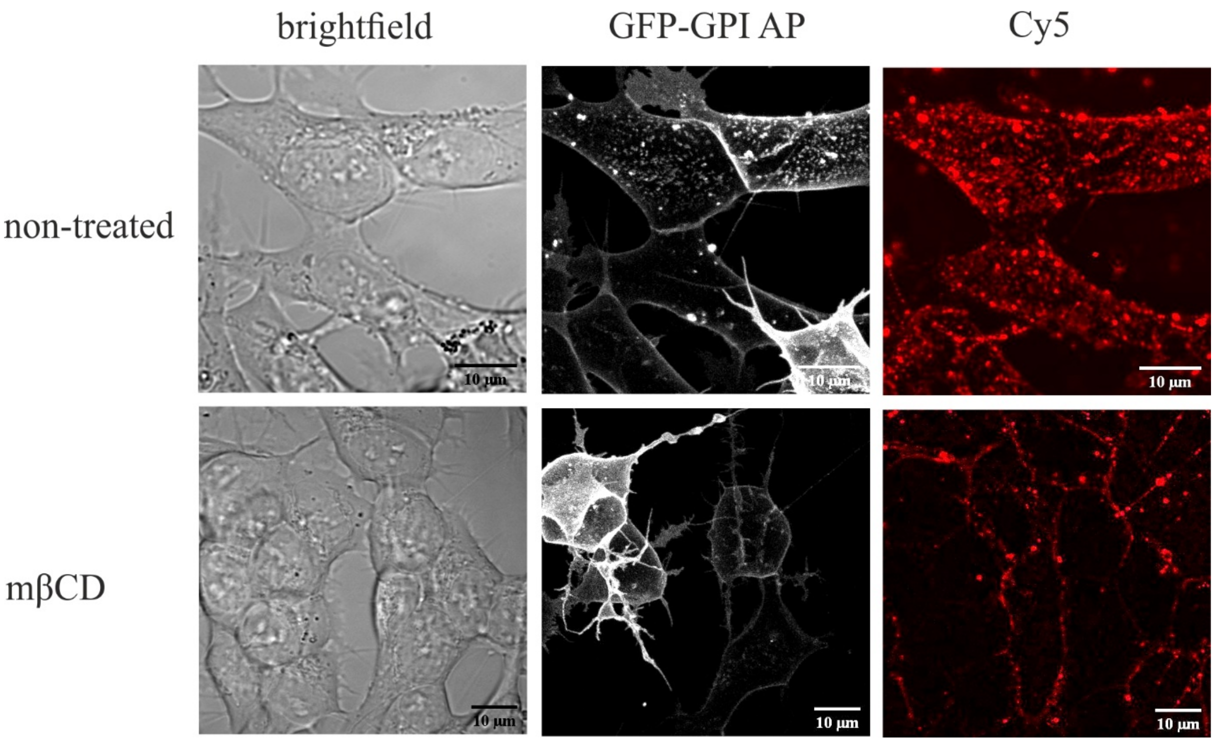

Figure S3. Cy5 labeled PrP-GPI incubated on GFP-GPI AP transfected SH-SY5Y cells non- and pre-treated with mβCD. Representative confocal images show GFP-GPI AP and Cy5 fluorescence with their respective brightfield channels after 5 min of PrP-GPI addition on non-treated cells (**top**), and on cells subjected to mβCD treatment (**bottom**) for 15-20 min, followed by PrP-GPI incubation for 40 min.

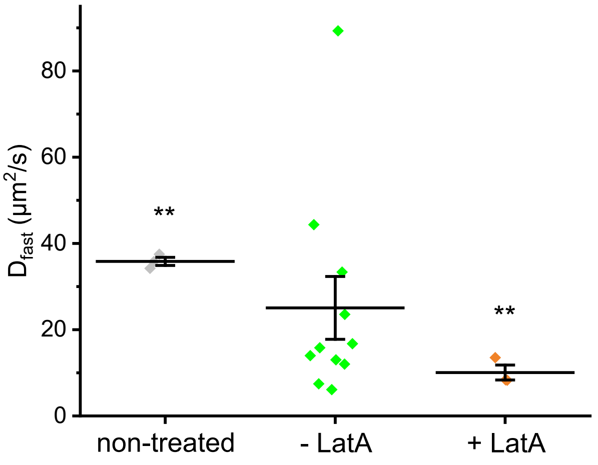

Figure S4. Average D values for the non-membrane-bound Cy5-labeled PrP-GPI observed close to the membrane of LifeAct-GFP transfected SH-SY5Y cells for non- and LatA-treated cells. PrP-GPI was measured before addition of LatA (-LatA) for 30 min, and after LatA treatment (+LatA) for 20-75 min. Control measurements were conducted on non-treated LifeAct-GFP cells (non-treated) for the same period of time of PrP incubation (120 min). Data are mean values ± SD of the number of ACFs (3 and 11) that are identical to the number of measurements (N_m) for confocal FCS. Changes seen here are not significant based on the two-sample t-test.

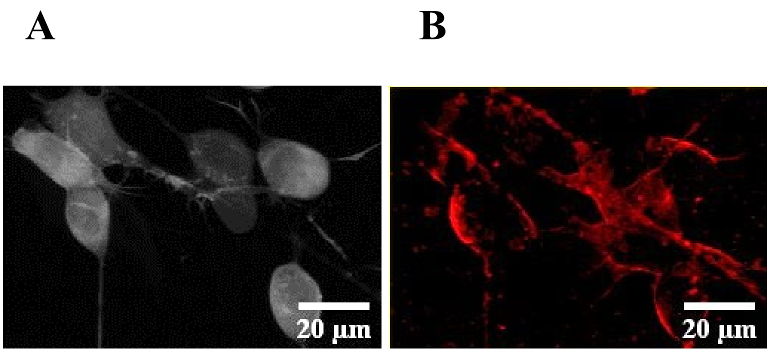

Figure S5. Cy5 labeled PrP-GPI incubated on LifeAct-GFP transfected SH-SY5Y cells. LifeAct-GFP SH-SY5Y cells are capable of expressing GFP labeled actin. Representative confocal images with LifeAct-GFP (**A**) and Cy5 fluorescence (**B**).

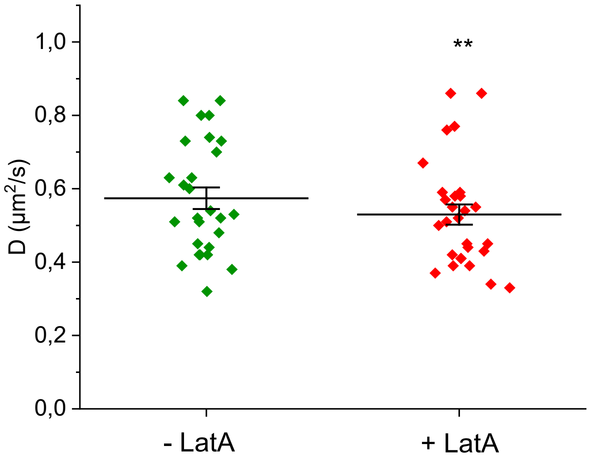

Figure S6. ITIR-FCS results of GFP fluorescence from GFP-GPI AP subjected to LatA treatment on SH-SY5Y cells. Data are mean values ± SD. ** indicates no significant difference.

Protein expression and purification

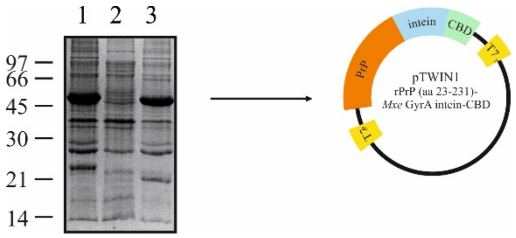

Figure S7. Following expression of PrP-Mxe GyrA intein-CBD fusion protein with SDS-PAGE analysis. Lane 2 shows rPrP (aa 23-231)-Mxe GyrA intein-CBD (MW: 51,156 Da) before induction with IPTG and lane 3 after 22 h of IPTG induction. Lane 1 shows the bands of the LMW marker.

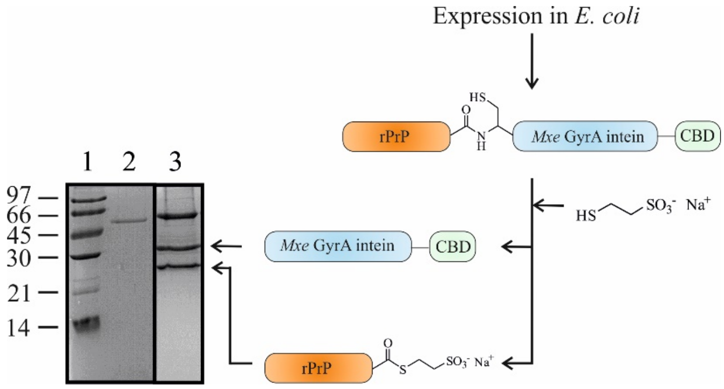

Figure S8. MESNA-mediated intein cleavage reaction followed by SDS-PAGE. rPrP (aa 23-231)-*Mxe* GyrA intein-CBD fusion protein (MW: 51,156 Da) in lane 2 subjected to MESNA-mediated intein cleavage condition (lane 3). Lane 3 shows formation of PrP-α-thioester (aa 23-231) (MW: 23,324 Da) and *Mxe* GyrA intein-CBD (MW: 27,938 Da). The reaction proceeded until 50% of α-thioester was formed in relation to the fusion protein. Percentage was determined based on the integrated intensity values of the bands from SDS-PAGE using Image Lab 5.1 software.

A **B**

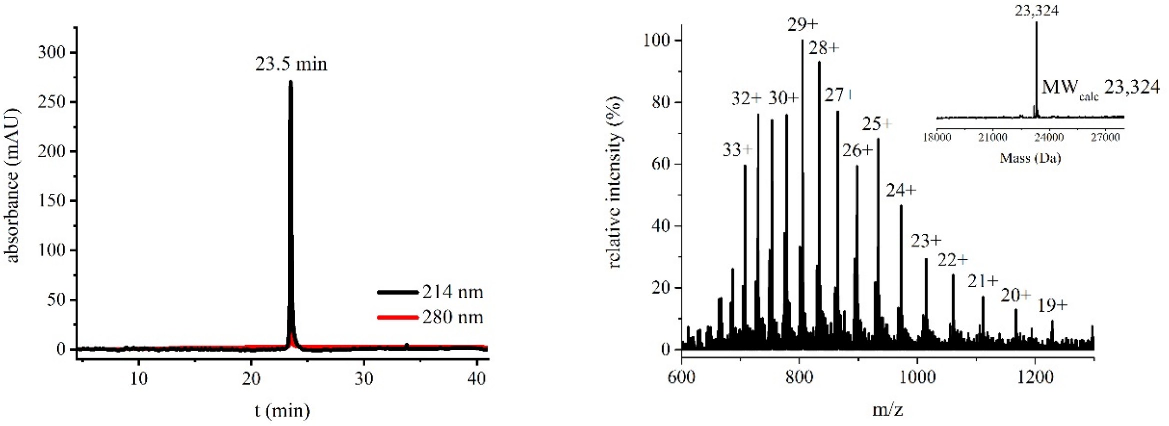

Figure S9. Characterization of PrP α-thioester. Analytical HPLC traces (**A**) (λ_{abs} = 214+280 nm, baseline corrected) and ESI-MS spectrum (**B**) of PrP-α-thioester (aa 23-231).

HPLC purification gave the PrP α-thioester (aa 23-231) in high purity, as can be seen in *Figure S9*. The deconvoluted mass spectrum shows a molecular weight of 23,324 Da in agreement with the calculated value. HPLC traces based on the detection of 214 and 280 nm absorption reveal single sharp peaks (*Figure S9*A). Isolation of the PrP α-thioester (aa 23-231) was achieved in a high yield of 12 mg/L *E. coli* medium.

Synthesis of fluorophore Cy5

Synthesis of Cy5 was accomplished in three steps starting from 2,3,3-trimethyl-3*H*-indolenine ***1***, an inexpensive precursor (*Scheme S1*). This approach afforded sufficient milligram amounts of the dye to use it in excess for coupling reactions in SPPS.

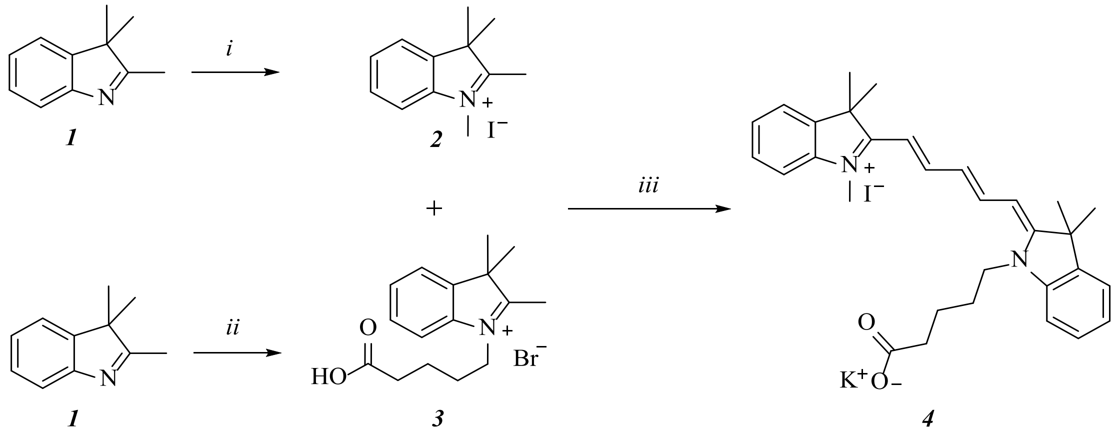

Scheme S1. Overview of Cy5 ***4*** synthesis. Reaction conditions: i = CH_3I, CH_2Cl_2 anh.; ii = 5-bromovaleric acid, KI, CH_3CN; iii = KOAc, Ac_2O, malonaldehyde bis(phenylimine) monohydrochloride, 1-butanol anh.

Two indolium parts were generated in reactions of the indolenine compound ***1*** with the corresponding halogenoalkanes in yields of 60 and 50% for ***2*** and ***3*** using protocols from Yamane *et al.*(57) and Zhang *et al.*(58), respectively. Although the linkage *via* the polymethine chain, according to a procedure from Korbel *et al.*,(59) gave 71 mg of pure Cy5 ***4***, this corresponds to only 6% isolated yield. The low yield is caused by a lack of selectivity of the reaction resulting in mixtures of methyl- (58) and acid-group modifed indolium (59) compounds to linked to themselves and to each other, respectively.

The purity of Cy5 ***4*** was assessed by HPLC and mass analysis (*Figure S10*), together with NMR spectroscopy (*Figure S13*).

A **B**

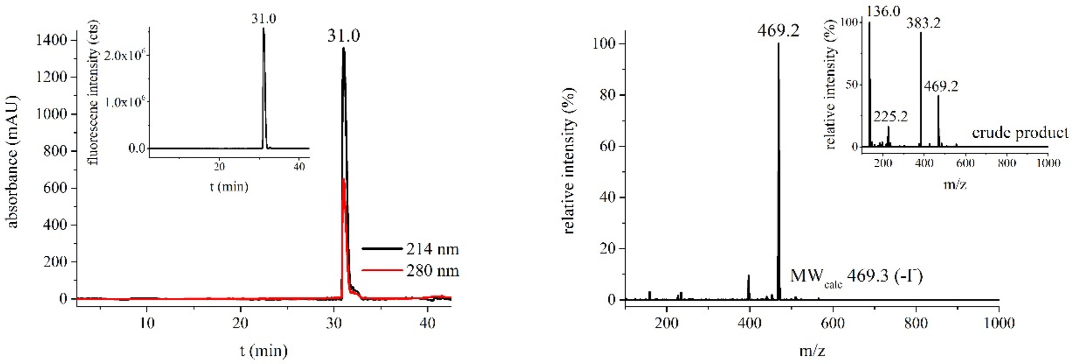

Figure S10. Characterization of Cy5 ***4***. Analytical HPLC traces (**A**) (λ_{abs} = 214+280 nm, $\lambda_{exc/em}$ = 630/650 nm, baseline corrected) and ESI-MS spectra (**B**) of crude and isolated Cy5 ***4***, respectively. The mass spectrum of the crude material shows a peak at m/z of 383.2 that can be assigned to two indolium ***2*** compounds bridged *via* the polymethine chain.

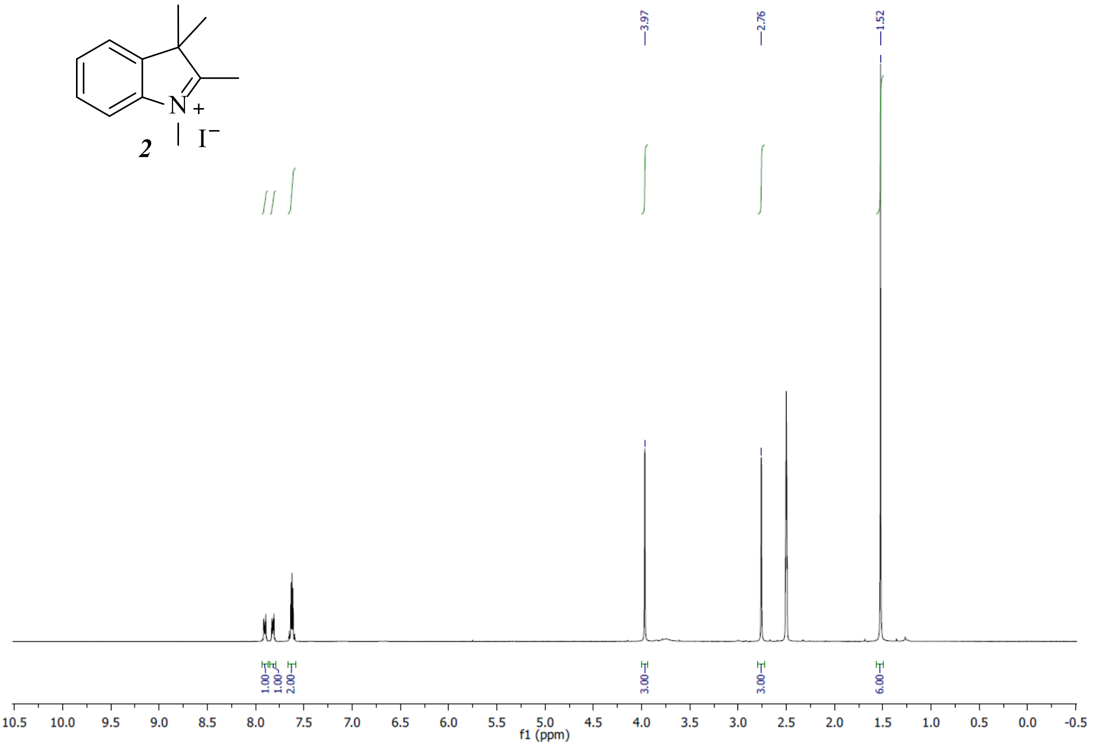

Figure S11. NMR spectrum of compound ***2***.

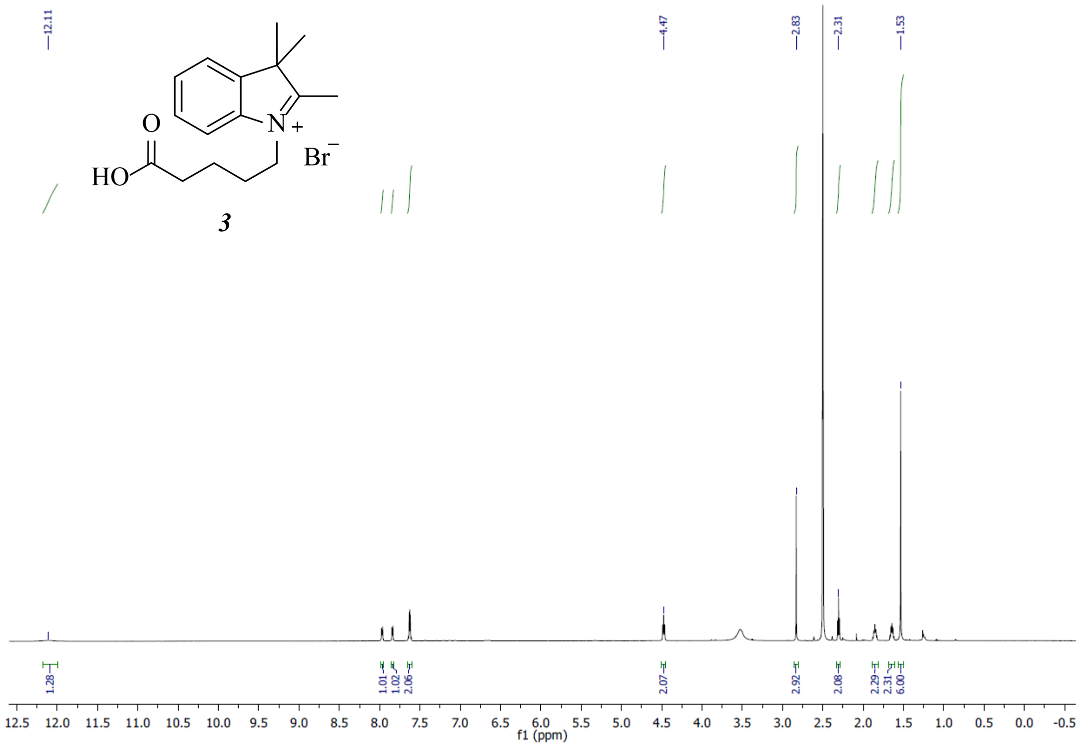

Figure S12. NMR spectrum of compound ***3***.

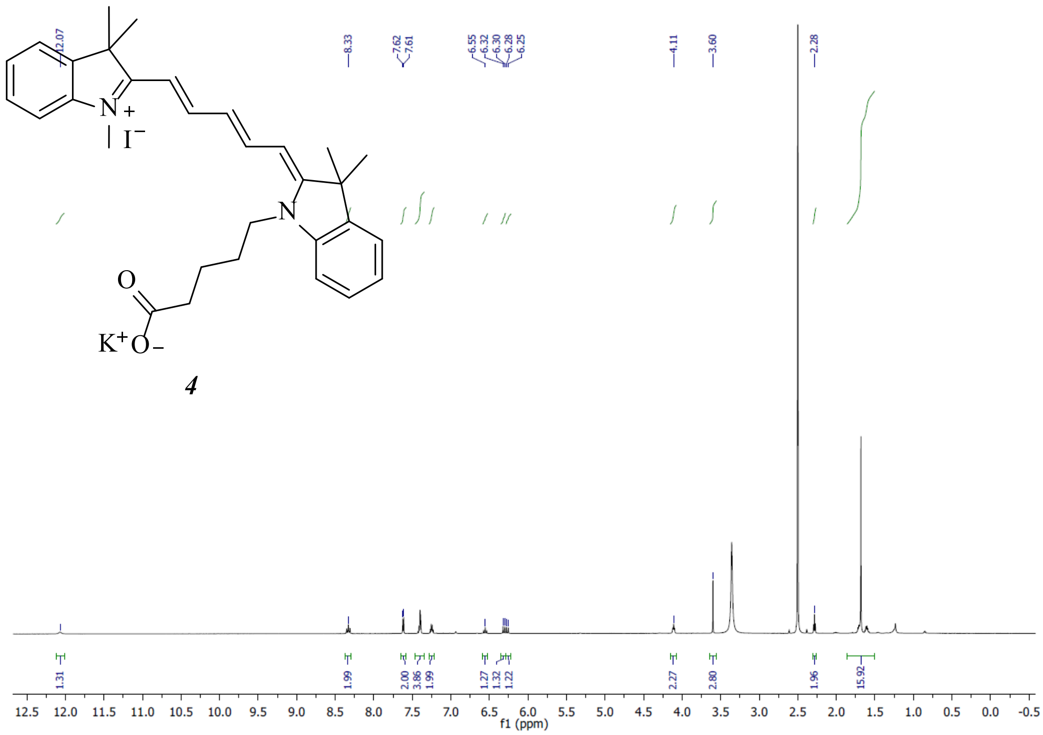

Figure S13. NMR spectrum of compound ***4***.

Solid phase peptide synthesis (SPPS)

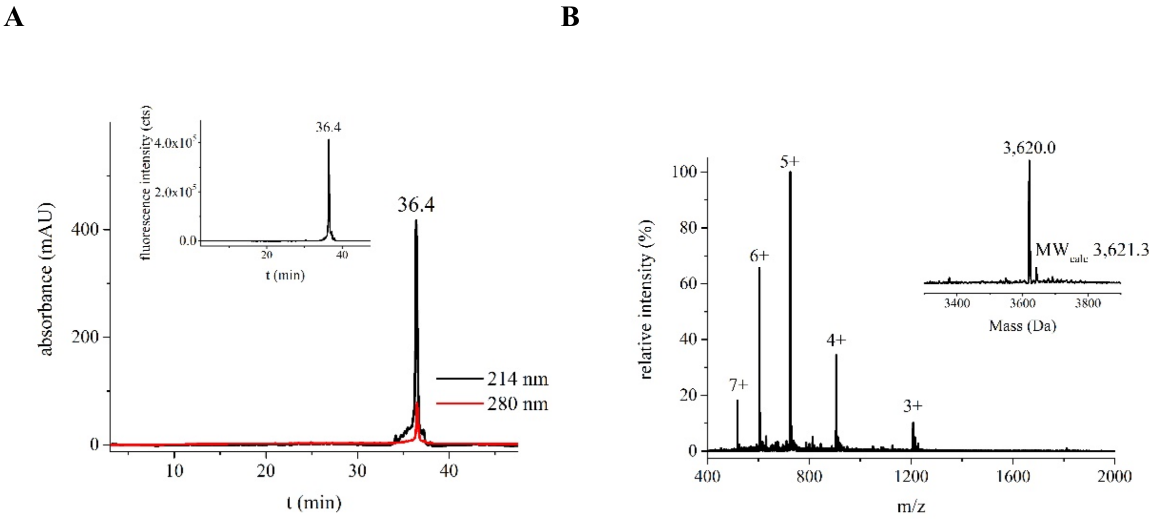

Figure S14. Characterization of Cy5 labeled GPI anchor-mimicking peptide. Analytical HPLC traces (A) (λabs = 214+280 nm, λexc/em = 630/650 nm, baseline corrected) and ESI-MS spectrum (B) of Cy5 labeled GPI anchor-mimicking peptide.

The applied SPPS strategy (Materials & Methods/Chemical Methods/SPPS) afforded milligrams of the required GPI-anchor building block in high purity and a yield of 21% (based on the amount of crude peptide) (Table S6). HPLC traces showed single sharp peaks with >95% purity. The mass spectrum could be deconvoluted to a molecular weight of 3,620.0 Da in agreement with the calculated value.

Expressed protein ligation (EPL)

The EPL reaction proceeded to a conversion of 56% formed Cy5 labeled PrP-GPI (Figure S15 and Table S7). HPLC purification afforded Cy5 labeled PrP-GPI in yields of 14%. The purity was assessed *via* SDS-PAGE (Figure S17) and analytical RP-HPLC (Figure S16A), showing a sharp band and single peaks with >95% purity. The mass spectrum (Figure S16B) could be deconvoluted to a molecular weight of 26,804.0 Da in agreement with the calculated value.

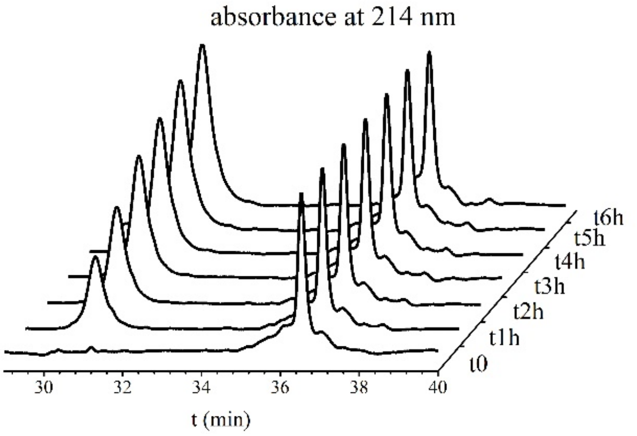

Figure S15. Following the EPL reaction between the PrP-α-thioester and Cy5 labeled peptide by HPLC. The ligation was monitored over 6 h *via* absorbance at 214 nm (baseline corrected and normalized). Peak areas of the formed Cy5 labeled PrP-GPI at t_R of 30.7 min increase with time proceeding and are accompanied by a decrease ascribed to Cy5 labeled GPI anchor-mimicking peptide at 36.5 min.

A B

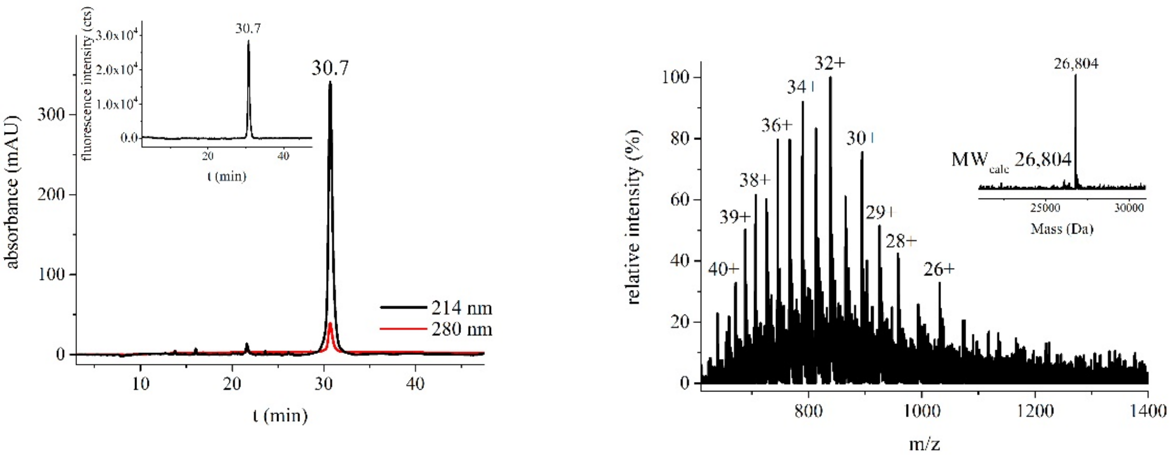

Figure S16. Characterization of Cy5 labeled PrP-GPI *via* HPLC and MS. HPLC traces (**A**) (λ_{abs} = 214+280 nm, inlay shows fluorescence detection at $\lambda_{exc/em}$ = 630/650 nm, baseline corrected) and ESI-MS spectra of Cy5 labeled PrP-GPI (**B**).

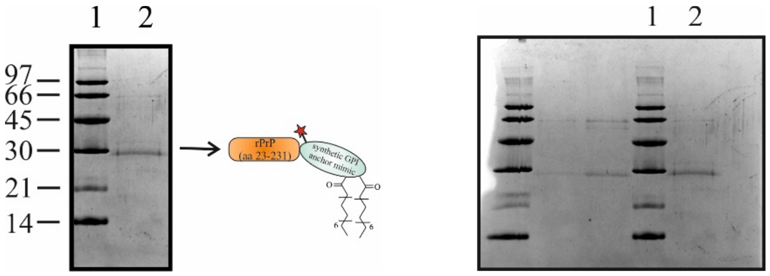

Figure S17. SDS-PAGE analysis of Cy5 labeled PrP-GPI. Cy5 labeled PrP-GPI (MW: 26,804 Da) analyzed with Coomassie-staining shows a single band in lane 2 next to the LMW marker (lane 1). SDS-gel (left) is cut out from whole gel (right).

Co-localization analysis of super-resolution (SR) images

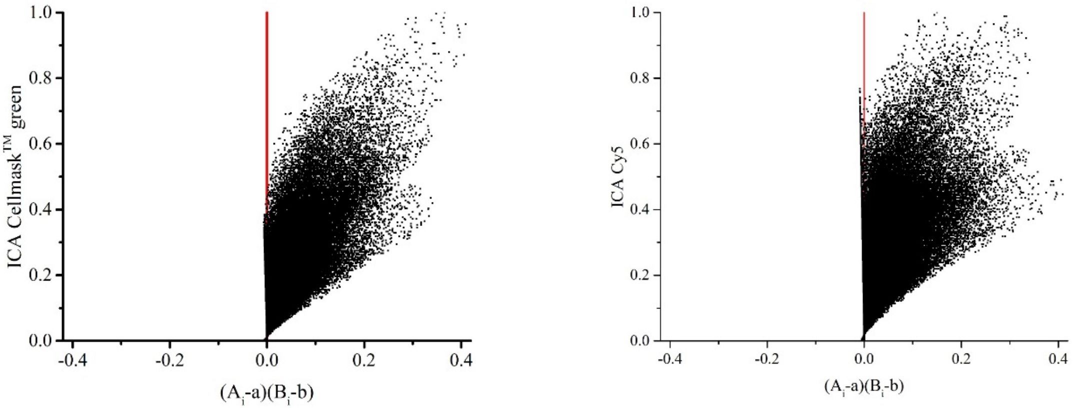

Figure S18. ICA plots of Cy5 labeled GPI anchor-mimicking peptide. Intensity correlation analysis (ICA) plots by Li (64) represent the co-localizing pixels of the Cellmask™ green fluorescence with Cy5 (left) and of the Cy5 fluorescence with Cellmask™ (right).

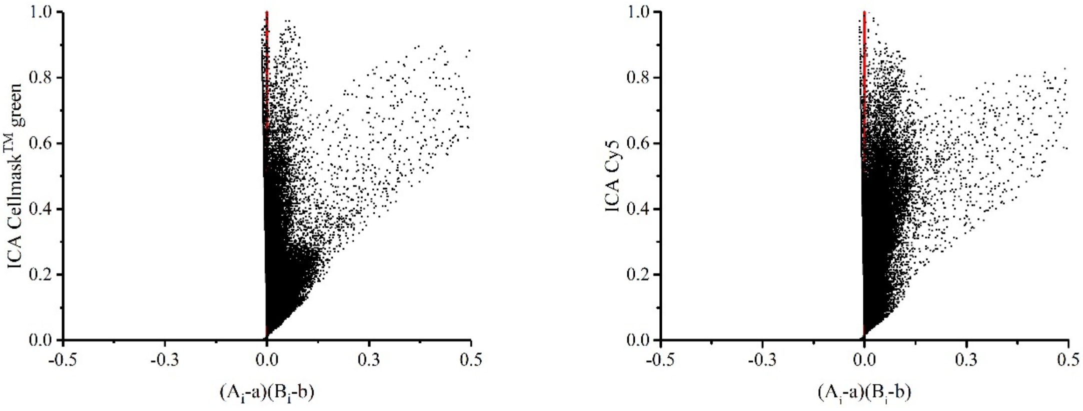

Figure S19. ICA plots of Cy5 labeled PrP-GPI. Intensity correlation analysis (ICA) plots by Li (64) represent the co-localizing pixels of the Cellmask™ green fluorescence with Cy5 (left) and of the Cy5 fluorescence with Cellmask™ (right).

Supporting Tables

Confocal imaging & fluorescence correlation spectroscopy (FCS)

GFP-GPI AP SH-SY5Y cells							
	PCC	*p*-value	*I*CQ	M_1	M_2	N_m	N_{cells}
non-treated	0.34 ± 0.052	100	0.16 ± 0.073	0.41 ± 0.304	0.41 ± 0.134	8	4
+ mβCD	0.14 ± 0.014*	100	0.07 ± 0.024	0.29 ± 0.456	0.30 ± 0.038	3	2

Table S1. Pearson's correlation coefficients (PCCs) for Cy5 labeled PrP-GPI and GFP-GPI AP subjected to mβCD. PrP accumulation on the cell membrane was compared over a time period of 40 min of PrP incubation for non-treated cells and cells subjected to mβCD for 20 min prior PrP addition. N_m corresponds to the amount of measurements. N_{cells} is the number of measured cells. Statistical significance is indicated by * (with p <0.05 based on the two-sample t-test) and ** indicates no significant difference.

GFP-GPI AP on SH-SY5Y cells							
	D_{slow} (μm^2/s)	D_{fast} (μm^2/s)	F_{slow}	τ_{trip} (μs)	F_{trip}	N_m	N_{cells}
non-treated	0.52 ± 0.019	18.25 ± 4.616	0.48 ± 0.047	23.4 ± 8.28	0.17 ± 0.029	2	2
+mβCD	0.61	17.73	0.55	26.8	0.20	1	1

Table S2. Confocal FCS results of the GFP fluorescence from GFP-GPI AP subjected to mβCD treatment. Data are mean values ± SD of the number of ACFs (2 and 1) that is identical to the number of measurements (N_m) for confocal FCS.

Cy5 labeled PrP-GPI on GFP-GPI AP SH-SY5Y cells					
	D_{slow} (μm^2/s)	D_{fast} (μm^2/s)	F_{slow}	N_m	N_{cells}
non-treated	0.60 ± 0.130	30.69 ± 12.877	0.36 ± 0.248	5	4
+mβCD	0.57 ± 0.106**	63.82 ± 24.480*	0.48 ± 0.230	5	4

Table S3. Confocal FCS results of the Cy5 fluorescence from PrP-GPI subjected to mβCD treatment. Data are mean values ± SD of the number of ACFs (64) that is identical to the number of measurements (Nm) for confocal FCS. Statistical significance is indicated by * (with p <0.05 based on the two-sample t-test) and ** indicates no significant difference.

Cy5 labeled PrP-GPI on LifeAct-GFP SH-SY5Y cells					
	D_{slow} (µm^2/s)	D_{fast} (µm^2/s)	F_{slow}	N_m	N_{cells}
non-treated	0.55 ± 0.151**	35.83 ± 1.638**	0.29 ± 0.120	3	2
-LatA	0.64 ± 0.107	25.06 ± 24.157	0.36 ± 0.164	11	7
+LatA	0.36 ± 0.120*	10.06 ± 3.009**	0.42 ± 0.287	3	2

Table S4. Confocal FCS results of the Cy5 fluorescence from PrP-GPI subjected to LatA treatment in LifeAct-GFP transfected SH-SY5Y cells. Data are mean values ± SD of the number of ACFs (3 and 11) that is identical to the number of measurements (N_m) for confocal FCS. Statistical significance is indicated by * (with p <0.05 based on the two-sample t-test) and ** indicates no significant difference.

GFP-GPI AP on SH-SY5Y cells		
	D (µm^2/s)	N_{cells}
non-treated	0.57 ± 0.153	27
+LatA	0.53 ± 0.146**	28

Table S5. ITIR-FCS results of GFP fluorescence from GFP-GPI AP subjected to LatA treatment on SH-SY5Y cells. Data are mean values ± SD and ** indicates no significant difference.

Solid phase peptide synthesis (SPPS)

peptide	synthesis scale (mmol)	Yield of				
		crude peptide		purified peptide		
		(mg)	(%)	(mg)	based on scale (%)	based on crude peptide (%)
Cy5 labeled	0.03	30	28	6.3	6	21

Table S6. Yields of GPI anchor-mimicking peptide obtained by SPPS.

Expressed protein ligation (EPL)

t (h)	integrated peak area at 214 nm (%)	
	Cy5 labeled PrP-GPI	Cy5 labeled peptide
0	0	100
1	37	63
2	45	55
3	51	49
4	54	46
5	56	44

Table S7. Following the EPL reaction between the PrP-α-thioester and Cy5 labeled peptide by HPLC. According to the absorbance at 214 nm the ligation is finished after 5 h with 56% of formed Cy5 labeled PrP-GPI. Integration of peak areas was accomplished with the software OriginPro using HPLC traces at 214 nm.

Materials

Molecular biology

If not stated otherwise, chemicals for buffer preparation were purchased from Sigma-Aldrich (Vienna, Austria), Fisher-Scientific (Vienna, Austria), VWR (Darmstadt, Germany) and Roth (Karlsruhe, Germany). Buffers were prepared using Milli-Q® water (dd H_2O). All plasmids were sequenced at Eurofin Genomics (Vienna, Austria) and Microsynth (Vienna, Austria). Chemical-competent cells were purchased from Merck Millipore (Darmstadt, Germany). Centrifugal filtration units used were Amicon Ultra-15 with a molecular weight cut-off (MWCO) of 30 and 10 kDa from Millipore (Darmstadt, Germany) and Vivaspin® 500 with MWCO of 3 kDa from Sartorius (Göttingen, Germany) or of 10 kDa from GE Healthcare (Freiburg, Germany). PD-10 Desalting Columns were purchased from GE Healthcare (Freiburg, Germany). Folding implements *N*-octyl-*β*-*D*-glucopyranoside (OG) and Slide-A-Lyzer™ cassettes with MWCO of 10 kDa were obtained from GERBU (Heidelberg, Germany) and Fisher-Scientific (Vienna, Austria). Low molecular weight (LMW) protein marker was purchased from GE Healthcare (Freiburg, Germany).

Chemicals

All chemical reagents and solvents were purchased in the highest available quality from Sigma-Aldrich (Vienna, Austria) or Fisher-Scientific (Vienna, Austria) if not noted otherwise.

Cell culture

SH-SY5Y cells were purchased from ATCC (Manassas, USA). Dulbecco's Modified eagle medium (DMEM) with high glucose content, its supplements fetal bovine serum (FBS), penicillin and streptomycin (PS) were obtained either from HyClone, GE Healthcare Life Sciences (South Logan, USA) and PAA Laboratories (Linz, Austria), or from GIBCO Invitrogen (Karlsruhe, Germany). 1× Phosphate buffered saline (PBS) without calcium and magnesium was purchased from BioWhittaker®, Lonza (Basel, Switzerland). 10× trypsin solution was from Sigma-Aldrich (St. Louis, USA). CellMask™ green plasma membrane stain together with live cell imaging solution was purchased from Fisher-Scientific (Vienna, Austria). 1× Hanks' balanced salt solution (HBSS) was from Life Technologies (Grand Island, USA). Green fluorescent protein-glycosylphosphatidylinositol anchored protein (GFP-GPI AP) was received as a kind gift from Dangerfield, J. from Anovasia Pte Ltd (Singapore) (78,79). For electroporation, electrolytic (E-) and resuspension (R-) buffer were from Invitrogen (Carlsbad, USA). LifeAct-GFP transfected SH-SY5Y cells were provided by the Wohland laboratory at NUS (Singapore) (80). Methyl-β-cyclodextrin (mβCD) and latrunculin A (LatA) were from Sigma-Aldrich (St. Louis, USA). Dyes for calibration Atto655 and Atto488 were purchased from Sigma-Aldrich (St. Louis, USA). Microscopy measurements were performed using Nunc™ Lab-Tek™ 8-well chambers (Permanox, #1.0 borosilicate coverglass) from Thermo Scientific (St. Louis, USA), No. 1.0 35 mm glass-bottom dishes from MatTek (Ashland, US) and μ-slide 8-well ibiTreat chambers

(#1.5 polymer coverslip, tissue culture treated) from ibidi (Martinsried, Germany). Pro-Ject™ protein transfection reagent kit was purchased from Pierce Biotechnology (Rockford, USA). Mounting medium Roti®-Mount FluorCare was obtained from Roth (Karlsruhe, Germany).
